# Supplementary material for: Shifting the gaze of the physician from the body to the body in a place: A qualitative analysis of a community-based photovoice approach to teaching place-health concepts to medical students
Source: PLoS One. 2020 Feb 11;15(2):e0228640. doi: 10.1371/journal.pone.0228640 (PMC7012448; doi:10.1371/journal.pone.0228640)
Supplement: S1 File — (DOCX) [file pone.0228640.s001.docx]

**S1 File: Exhibitions, Video, and GIS Maps**

**“Imagining the West Side: Constructing Health through the Built Environment”**

- The panels and graphics for the Exhibition were produced by Tyler Williams a graphic designer living in Parkersburg, West Virginia.
- The GIS maps were produced by Matthew Purtill, second author of the paper, a lecturer at SUNY Freedonia Geosciences Department, with an expertise in Prehistoric Archaeology, and graduate of West Virginia University in Geography at the time of this project.
- The photos, captions, and reflections from the medical students, faculty, and neighborhood residents along with the GIS maps were curated into an exhibition.
- The video recorded interviews and photos with the medical school and community-based participants were edited into a forty-minute video at <https://researchrepository.wvu.edu/faculty_publications/1104>
- The exhibition and video served to:
  - Demonstrate how medical students and faculty members integrated concepts about the West Side community as a place that shaped the lives of its’ members whom they saw in the health clinics;
  - Integrate the voices of vulnerable groups into public policy decision-making; and
  - Shed light on the built environment and population health status of those that lived on the west side of Charleston, West Virginia.

**Three Exhibitions Venues and Dates**

- West Virginia University, Robert C. Byrd Health Sciences Center April 10-27, 2017: a guided tour by the lead researcher and panel discussion featuring WVU School of Medicine Charleston students and faculty plus community members.
- West Virginia University, Downtown Campus Library August 7-31, 2017
- Lecture and Exhibition: Marcy C. Snow Elementary Charleston, West Virginia September 11-14th, 2017

**Gazette News Article: Photos: West Side project unveiled at Charleston school September 12, 2017**

[https://www.wvgazettemail.com/news/photos-west-side-project-unveiled-at-charleston-](https://www.wvgazettemail.com/news/photos-west-side-project-unveiled-at-charleston-school/article_3c1059d7-2fe9-508f-919e-d2b3a77c4de3.html) [school/article_3c1059d7-2fe9-508f-919e-d2b3a77c4de3.html](https://www.wvgazettemail.com/news/photos-west-side-project-unveiled-at-charleston-school/article_3c1059d7-2fe9-508f-919e-d2b3a77c4de3.html)

**Poster for Exhibition at West Virginia University Health Sciences Center**


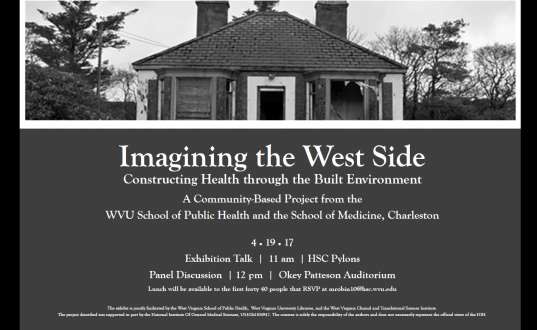


**Press Release**

CONTACT: Sally Deskins, Exhibits & Programs Coordinator, WVU Libraries 304.293.0369; [sbdeskins@mail.wvu.edu](mailto:sbdeskins@mail.wvu.edu)

**Downtown Campus Library hosts ‘Imagining the West Side’ exhibit, tour and talk in conjunction with Campus Read programming**

MORGANTOWN, W.Va. – [West Virginia University](http://www.wvu.edu/) [Libraries](https://lib.wvu.edu/) and the [WVU School of](http://publichealth.hsc.wvu.edu/) [Public Health](http://publichealth.hsc.wvu.edu/) are collaborating on an exhibit and talk that examines the impact of socio- economic factors on health issues and connects with the [University’s 2017-2018](http://campusread.wvu.edu/) [Campus Read title, *Hidden Figures*](http://campusread.wvu.edu/)*. Hidden Figures* tells the story about three African- American women at NASA—two from West Virginia—and their experience with discrimination in mid-twentieth century America.

*Imagining the West Side: Constructing Health through the Built Environment*, on display at the [Downtown Campus Library](https://lib.wvu.edu/downtown/) Atrium Aug. 7-31, presents a 21st century account of marginalization, population health, economic development and geography specifically with regard to Charleston, W.Va.’s West Side. Dr. Lauri Andress, whose work is visualized in *Imagining the West Side* exhibition, will provide a narrated tour of the exhibition at 11 a.m. Aug. 24 starting in the DCL Atrium.

“Today health is determined by where we live work and play. But what happens to the health of groups when their community, after years of disinvestment and neglect, is finally restored but the residents can no longer afford the costs to live there?” asks Andress, an assistant professor in the Department of Health Policy, Management, and Leadership in the WVU School of Public Health.

Andress explains: “*Imagining the West Side* explores an overlooked form of discrimination that happens when stigmatized groups are marginalized through geography starting with limitations on places where they are allowed to buy property, followed by decades of community neglect and disinvestment, and ending with economic development that transforms the space into a place where the residents are slowly, culturally and economically priced out and displaced by higher income residents.”

Incorporating participatory photoanalytics, GIS mapping, and video with an aim of integrating the voices of vulnerable groups into public policy decision-making, the exhibit sheds light on the built environment and population health status of Charleston’s west side. Community members and WVU School of Medicine students provided photos and narratives. The exhibition closes with suggested policy solutions to help ensure equitable development.

Following the exhibit tour, comments will be delivered by two individuals who have first- hand experience with Charleston’s West Side, Pastor Matthew Watts, CEO of HOPE Community Development Corporation (CDC), and David Fryson, a Vice President for West Virginia University and head of the Division of Diversity, Equity and Inclusion.

Pastor Watts is senior pastor of Grace Bible Church on the West Side, and founded HOPE Community Development Corporation, with the mission of empowering the residents on the West Side through spiritual renewal, education, training, employment, and economic development.

The presentation is part of WVU Campus Read programming as it relates to the health of marginalized individuals like the women in *Hidden Figures* and the residents of

Charleston and beyond. An exhibition video is available [online](http://mediaspace.hsc.wvu.edu/media/Imagining%2BThe%2BWest%2BSide/1_673dsywc/42739741). The exhibit is jointly facilitated by the WVU School of Public Health, WVU Libraries, and the West Virginia Clinical and Translational Science Institute, and supported in part by the National Institute of General Medical Sciences.

Cutline: Dr. Andress giving a tour at the spring exhibition at the WVU Health Sciences Center.

-WVU-mm/07/28/17


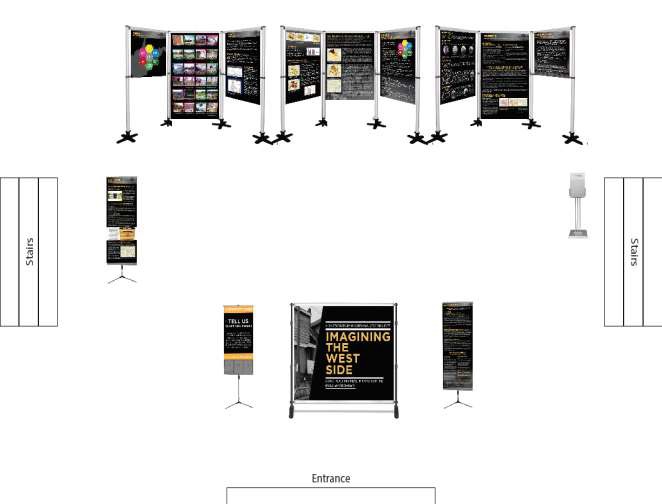
**Exhibition Schematic**

**Exhibition Panels**

**West Virginia University, Robert C. Byrd Health Sciences Center April 10-27, 2017**

**Background Panel**


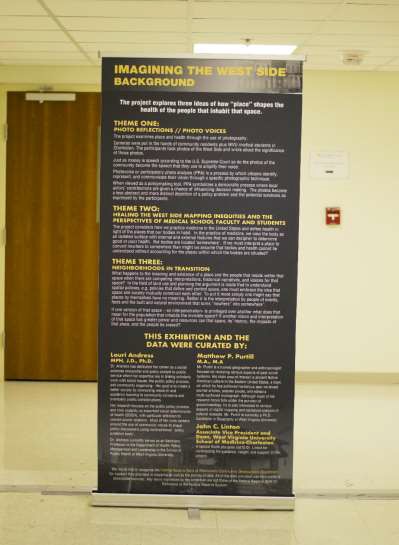


The Exhibition explored three ideas of how “place” shapes the health of the people that inhabit that space. Through the use of photography, the exhibition becomes a less abstract and more distinct depiction of a policy problem and the potential solutions as expressed by the study participants. The project put cameras in the hands of community residents plus West Virginia University medical students in Charleston. The participants took photos of the West Side and wrote about the significance of those photos.

**Theme 1 and 2**

Photos and Captions from Community Members and Mapped Data Describing the West Side


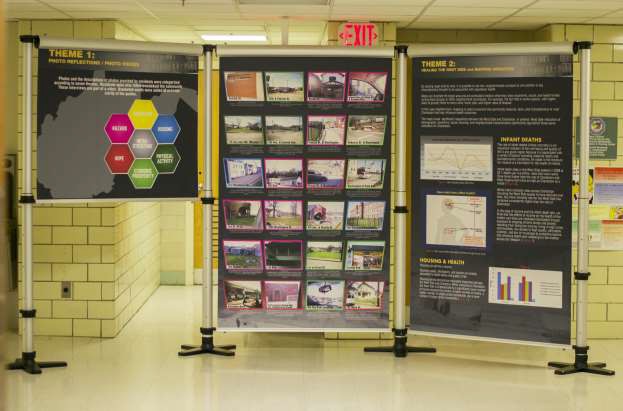


In the case of the exhibition all photos and descriptive captions were first reviewed by an external party, a graphic artist, who did not participate in the photo analytic workshops. This graphic artist placed photos into thematic categories. The lead researcher (LA) than reviewed the graphic artists’ coding of the photos in comparison to themes generated during the second community meeting of the study participants when they reviewed photos in April 2016. Themes were added, modified, and color coded by the lead researcher. The photos were then placed into the exhibition using color coded themes (Photo 1):

- Education/yellow
- Infrastructure/gray
- Hazard/pink
- Economic Prosperity/dark green
- Physical Activity/light green,
- Hope/red
- Housing/blue

**Theme 2**

Mapped Data and Photos, Narratives of Medical Students


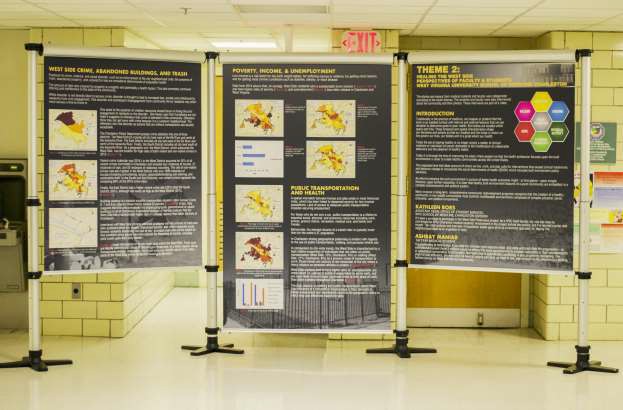


Mapping Inequities And The Perspectives Of Medical School Faculty And Students

The Exhibition considered how we practice medicine in the United States and define health in light of the places that our bodies in habit. By placing maps side by side, it is possible to see how neighborhoods compare to one another in key characteristics thought to be associated with population health. Maps can illustrate the target area and are particularly helpful when they show economic, social, and health trends across block groups or other neighborhood boundaries. For example, the fact that in some regions with higher rates of poverty there is more crime, fewer jobs, and higher rates of disease. In this case neighborhood mapping is used to examine the community features, facts, and characteristics in west Charleston that may influence health outcomes. The maps reveal significant inequalities between the West Side and Charleston. In general, West Side indicators of demographic, economic, social, housing, and neighborhood characteristics significantly lag behind those same indicators for Charleston.

**Theme 2 and 3**

Photos and Captions from Medical Students, Gentrification and Community Development


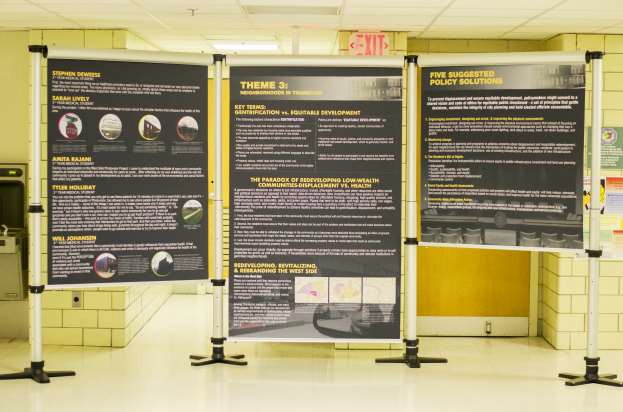


The Paradox of Redeveloping Low-Wealth Communities-Displacement Vs. Health

A government’s decisions on where to put infrastructure, transit, affordable housing, and other resources are often social and political decisions as opposed to fact based, data driven decisions. New investments can have positive impacts on neighborhood residents and health by providing opportunities for affordable housing, shopping, high quality schools, and infrastructure such as sidewalks, parks, and green space.

Places that tend to be older, with high poverty rates, low wages, high unemployment, and mostly multi- family or rental housing face a quandary in the effort to redevelop their community. The price of redevelopment to achieve healthy amenities can lead to gentrification, displacement, and unhealthy consequences.

Suggested Policy Solutions

To prevent displacement and ensure equitable development, policymakers might commit to a shared vision and code of ethics for equitable public investment - a set of principles that guide decisions, maintain the integrity of city planning and hold elected officials accountable.

**Evaluation and Feedback Panel with References**


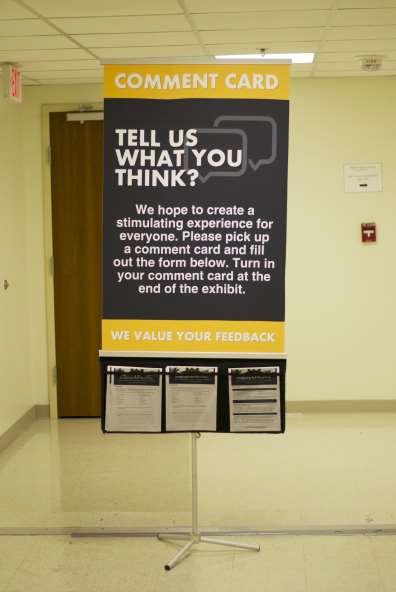


WEST SIDE OF CHARLESTON:

DEMOGRAPHIC, HOUSEHOLD, NEIGHBORHOOD, TRANSPORTATION, EDUCATION, AND INCOME DATA

Contents

[Introduction 16](#_TOC_250014)

[Demographic Trends 21](#_TOC_250013)

[Education Trends 29](#_TOC_250012)

[Employment 37](#_TOC_250011)

[Travel Time to Work 41](#_TOC_250010)

[Primary Mode of Transportation to Work 45](#_TOC_250009)

[Crime Statistics 49](#_TOC_250008)

[Insurance Rates 51](#_TOC_250007)

[Trends in Housing 52](#_TOC_250006)

[Neighborhood Characteristics 59](#_TOC_250005)

[Greenspace 59](#_TOC_250004)

[West Side City Trash Can Locations 60](#_TOC_250003)

[Discussion 62](#_TOC_250002)

[Conclusions 67](#_TOC_250001)

[Bibliography 69](#_TOC_250000)

Figures

Figure 1. General view of Charleston showing Census Tract boundaries 17

Figure 2. Shaded, numbered, areas represent *safe neighborhood canvassing zones* as designated by the Charleston Main Streets organization 18

Figure 3. West Side of Charleston neighborhood boundaries as defined by HOPE CDC. 19

Figure 4. Expanded view of the West Side, Census Tracts 1, 7, and 8, showing location of Census Tracts and Block Groups 20

Figure 5. Total population density of the City of Charleston, by Block Group 22

Figure 6. Age profile comparison for West Virginia, Charleston, and West Side 22

Figure 7. Population density and distribution of Charleston residents between the ages of birth and 21, by Block Group 23

Figure 8. Population density and distribution of Charleston residents between the ages of 22 and 44, by Block Group 24

Figure 9. Population density and distribution of Charleston residents between the ages of 45 and 64, by Block Group 25

Figure 10. Population density and distribution of Charleston residents above the age of 65, by Block Group 26

Figure 11. Distribution of Charleston residents self-identified as “African-American”, by Block Group ... 27 Figure 12. Distribution of Charleston residents self-identified as “Mixed-Race”, by Block Group 28

Figure 13. Location of middle and high schools within the Kanawha County school district. Stonewall Jackson Middle School and Capital High School are labeled and discussed in text 31

Figure 14. 2012-13 middle school math and reading proficiency rates per school attendance zone in Kanawha County, West Virginia. Percentages represent proportion of students that scored at, or above, mastery in reading and math. Attendance zone boundaries derived from the Department of Education’s National Center for Education Statistics (NCES), School Attendance Boundary Survey (SABS) 2013-2014. School performance data derived from the West Virginia Department of Education website (https://wvde.state.wv.us/boe/) for the 2012-13 academic year. 32

Figure 15. 2012-13 high school math and reading proficiency rates per school attendance zone in Kanawha County, West Virginia. Percentages represent proportion of students that scored at, or above, mastery in reading and math. Attendance zone boundaries derived from the Department of Education’s National Center for Education Statistics (NCES), School Attendance Boundary Survey (SABS) 2013-2014. School performance data derived from the West Virginia Department of Education website (https://wvde.state.wv.us/boe/) for the 2012-13 academic year. 33

Figure 16. Distribution of residents above the age of 25 who obtained a high school diploma, or GED equivalent, by Block Group 34

Figure 17. Results of the Getis-Ord Gi* statistic (standard deviation) calculation showing the distribution of “hot” and “cold” spots across Charleston in relation to high school diploma obtainment, by Block Group 35

Figure 18. Distribution of Charleston residents with either a 2-year, or 4-year, college degree, by Block Group 36

Figure 19. Medium annual income of Charleston residents, by Block Group 38

Figure 20. Average median income comparison for West Virginia, Charleston, and West Side 39

Figure 21. Percentage of Charleston residents between the ages of 20 and 64 living below the poverty line, by Block Group 39

Figure 22. Unemployment rate for Charleston residents above the age of 16 and participating within the civilian labor force, by Block Group 40

Figure 23. Civil work force unemployment rate by age group for Charleston 40

Figure 24. Map showing percent of citizens that travel less than 30 minutes to work, by Block Group ... 42

Figure 25. Map showing percent of citizens that travel between 30 and 59 minutes to work, by Block Group 43

Figure 26. Map showing percent of citizens that travel greater than 59 minutes to work, by Block Group

.................................................................................................................................................................... 44

Figure 27. Percentage of citizens that rely on public transportation (excluding taxi service) as primary mode of transportation for work, by Block Group 46

Figure 28. Percentage of citizens that rely on private motorized vehicles for primary mode of transportation for work, by Block Group 47

Figure 29. Percentage of citizens that rely on walking as primary mode of transportation for work, by Block Group 48

Figure 30. Distribution of 2015 violent and non-violent crimes for Charleston by crime district. 50

Figure 31. Percentage of Charleston residents under the age of 65 without healthcare insurance, by Block Group 51

Figure 32. Percentage of vacant buildings, by Block Group 53

Figure 33. Percentage of households occupied by owner, by Block Group 54

Figure 34. Percentage of households rented to non-owners, by Block Group 55

Figure 35. Percentage of households occupied by a single person, by Block Group 56

Figure 36. Percentage of households occupied by a single family unit, by Block Group 57

Figure 37. Percentage of households occupied by two, or more, family units, by Block Group 58

Figure 38. The location, and relative proportion, of Green Space within Charleston. Data on Green Space locations provided by City of Charleston, Planning Office 60

Figure 39. Distribution of city maintained trash cans within the West Side, specifically Census Tracts 7 and 8. Locational data provided by City of Charleston 61

Figure 40. Comparison of Charleston and West Side racial composition 63

Figure 41. High school and college diploma obtainment rates for Charleston and the West Side 63

Figure 42. Comparison of Charleston and West Side factors for work travel times and means of work transportation 64

Figure 43. Comparison of unemployment rates and percentage of population below poverty line for Charleston and the West Side 65

Figure 44. Comparison of building vacancy rates and percentage of people without health insurance for the West Side and Charleston 66

Figure 45. Comparison of housing trends between West Side and Charleston 66

Figure 46. Comparison of relative percentage of green space by Block Group for the West Side and Charleston 67

Figure 47. Comparison of crime statistics between West Side (West District) and the remainder of Charleston (South and East Districts combined) 67

# Introduction

The West Side of Charleston, or simply the West Side, consists of approximately 15 square miles of land north of the Kanawha River and west of the Elk River. This includes portions of Census Tracts 1, 2, 3, 5, 6, 7, and 8 (Figure 1). Based on U.S. Census Bureau’s 2014 American Community

Survey 5-year Estimates, Tracts 1, 2, 3, 5, 6, 7, and 8 includes 16,549 residents, or approximately 33 percent of the total population for the city of Charleston.

Among Charleston residents, this area can be further subdivided into various neighborhoods or communities, the exact boundaries of which are contested among residents, city officials, and revitalization organizations. For example, as part of their Neighborhood Branding Initiative, Charleston’s West Side Main Streets organization recognizes 16 distinct Safe Neighborhood Canvassing Zones some of which align with traditional historic West Side neighborhoods (Figure 2). In contrast, Pastor Matthew Watts of HOPE CDC discusses the West Side more broadly and suggests that it roughly consists of five neighborhoods: North Charleston (Census Tracts 1 and 2), Edgewood (Census Tract 2), West Side Hills (Census Tract 6), Garrison Avenue Area (Census Tract 5), and the West Side Flats (Census Tracts 7 and 8) (Figure 3).

Of this broader area, Census Tracts 1, 7, and 8 represent the geographical, and cultural, center of the West Side and is the focus of various revitalization and safety initiates spearheaded by organizations such as HOPE CDC, Keep Your Faith Corporation, West Side Main Street, West Side Neighborhood Association, and the Charleston Urban Renewal Authority (CURA), among others. For the purpose of this project, the term ‘West Side’ is restricted to communities within the seven Block Groups nested within Census Tracts 1, 7, and 8 (Figure 4). This includes parcels bordered by Washington St. to the north, the Kanawha River to the south, Keller Dr. to the west, and the Elk River to the east. By this definition, the West Side includes what HOPE CDC refers to as portions of the North Charleston and West Side Flats neighborhoods and corresponds to West Side Main Street’s Safe Neighborhood Canvassing Zones 5, 6, 7, 8, 9, 13, 14, 15, and 16.

Various sources were used to generate demographic, household, economic, neighborhood, educational, and city infrastructure data for the West Side, the City of Charleston as a whole, and the State of West Virginia. Much of the initial compilation of data and early mapping for this project was conducted by the Federal Reserve Bank of Richmond’s Community Development Department1. Unless otherwise noted, all data presented in this documented, including mapped data, has derived from the U.S. Census Bureau’s 2014 American Community Survey 5-year Estimates. As discussed below, the emergent profile of the West Side illustrates its unique character and points of departure from the remaining Charleston neighborhoods, as well as the state of West Virginia.

1 All data provided by the Federal Reserve was from publically assessable sources. Any views expressed by West Virginia University in the report are not those of the Federal Reserve Bank of Richmond or the Federal Reserve System.


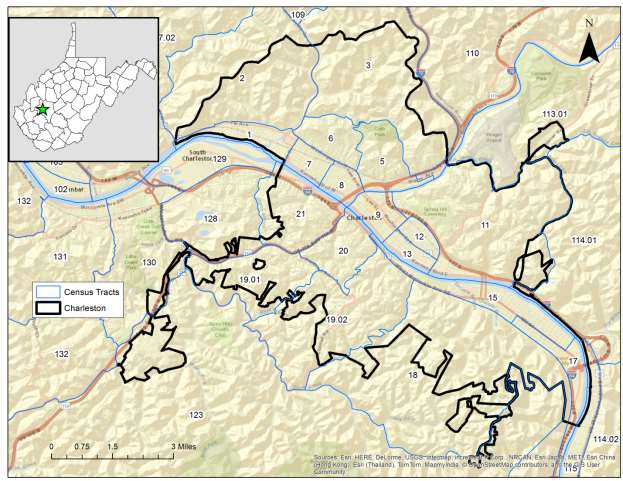


**Figure 1. General view of Charleston showing Census Tract boundaries**


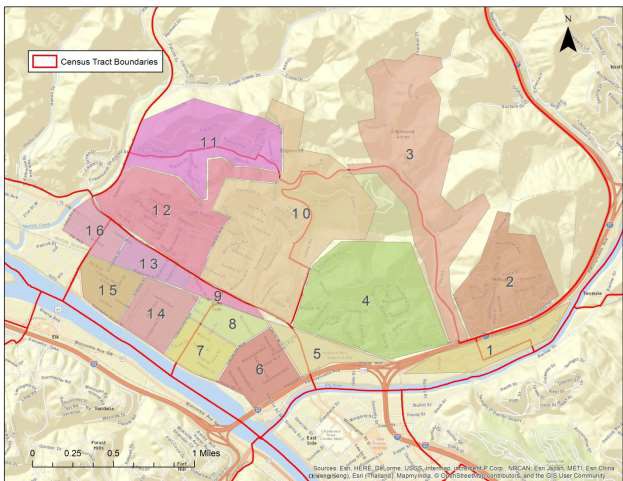


**Figure 2. Shaded, numbered, areas represent *safe neighborhood canvassing zones* as designated by the Charleston Main Streets organization**


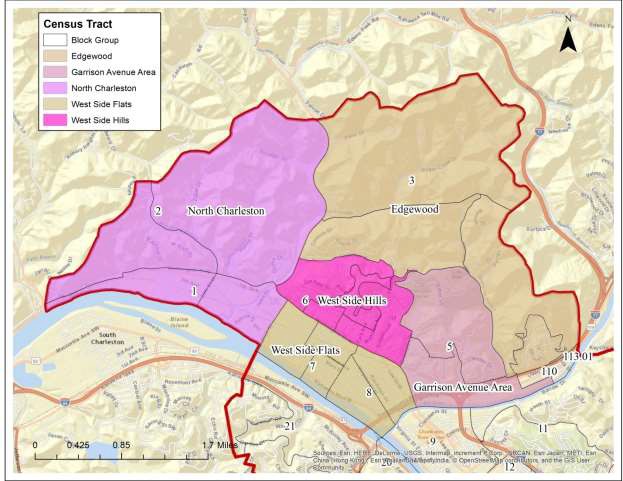


**Figure 3. West Side of Charleston neighborhood boundaries as defined by HOPE CDC.**


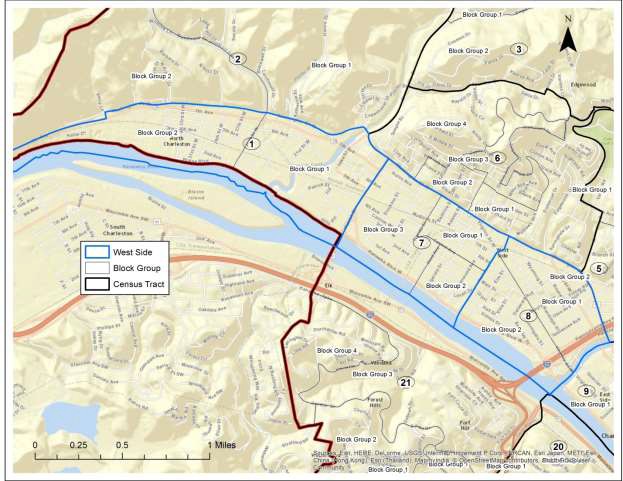


**Figure 4. Expanded view of the West Side, Census Tracts 1, 7, and 8, showing location of Census Tracts and Block Groups**

# Demographic Trends

Of Charleston’s 50,911 residents, 4779, or 9%, live in West Side communities (Figure 5). Demographic data for West Side indicates a population consisting of a substantial African- American and mixed-race population (27.4% and 18%, respectively), a slightly higher ratio of females to males (53% to 47%, respectively), and a roughly uniform age profile with the possible exception of the ‘children under 5 years of age’ category which is slightly over-represented in West Side (Figure 6). A second view of the Charleston age profile is presented geographically in Figures 7 through 10. These figures display Charleston residents grouped into four age brackets (0-21; 21-44; 45-64; and 65+). One interesting trend can be seen in Block Group 3 of Census Tract 7 where a low population base is characterized by a comparatively high proportion of elderly citizens (19% of the block population). This area represents one of the largest geographic concentrations of elderly citizens, as measured in block units, found anywhere in the city.

As mentioned, the West Side appears more racially diverse than other parts of the city or regions of the state. Although Charleston is predominately white (81%), the West Side has a substantial African-American population that accounts for 25% of the population in Census Tracts 1, 7, and 8 (Figure 11). The West Side also contains a relatively high percentage (19.3%) of people that self-identified as ‘mixed race’ (two or more races) (Figure 12). The mixed-race proportion for the West Side especially is notable given the low percentages reported for the city as a whole (6.6%) and the state of West Virginia (2%).

To statistically test this apparent pattern, a *two proportion Z-test* was calculated comparing the relative proportions of African-American, white, and mixed race percentages between the West Side and Charleston2. Test results were significant (*Pearson* χ2= 4604.261, *df* = 2, *p*<0.001) which indicates that the West Side has a statistically significant clustering of African-American and mixed-race populations when compared to the remainder of the city.

2 In cases of statistical tests calculated as part of this research, “West Side” was defined as Census Blocks 1, 7, and 8; whereas “Charleston” was defined as all city areas excluding Census Blocks 1, 7, 8 (e.g., Charleston excluding the West Side). This approach differs from other sections of this report where discussion of “Charleston” typically includes all Census Blocks within city limits.


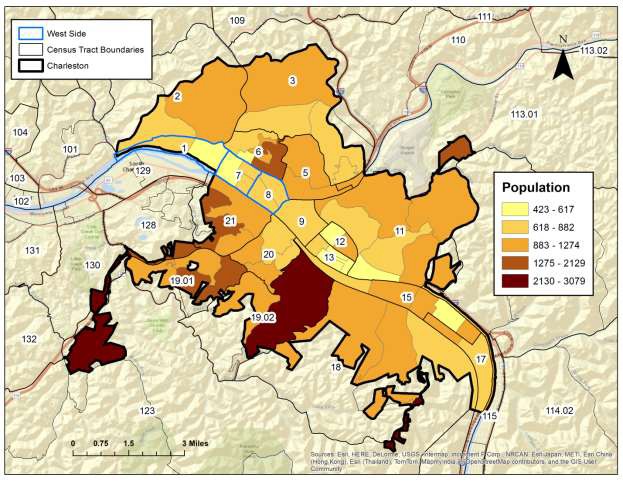


**Figure 5. Total population density of the City of Charleston, by Block Group**


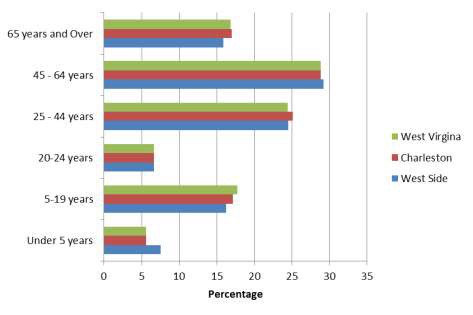


**Figure 6. Age profile comparison for West Virginia, Charleston, and West Side**


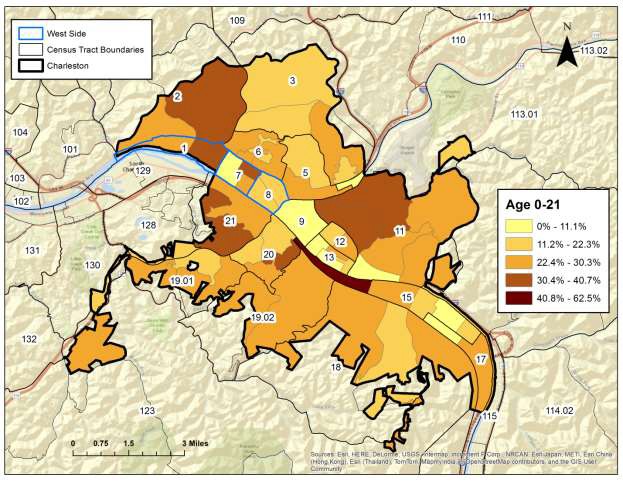


**Figure 7. Population density and distribution of Charleston residents between the ages of birth and 21, by Block Group.**


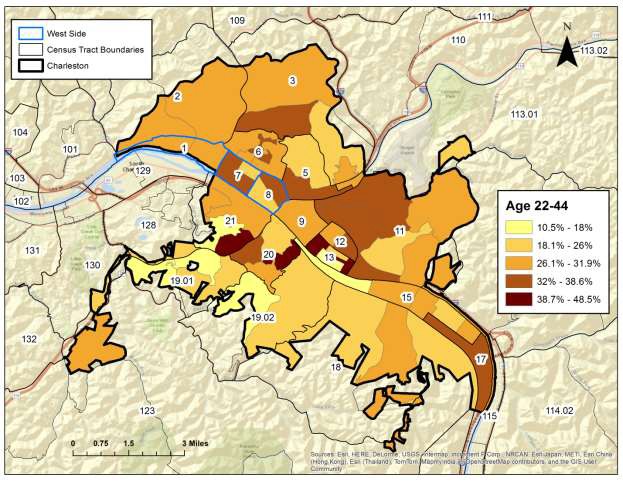


**Figure 8. Population density and distribution of Charleston residents between the ages of 22 and 44, by Block Group**


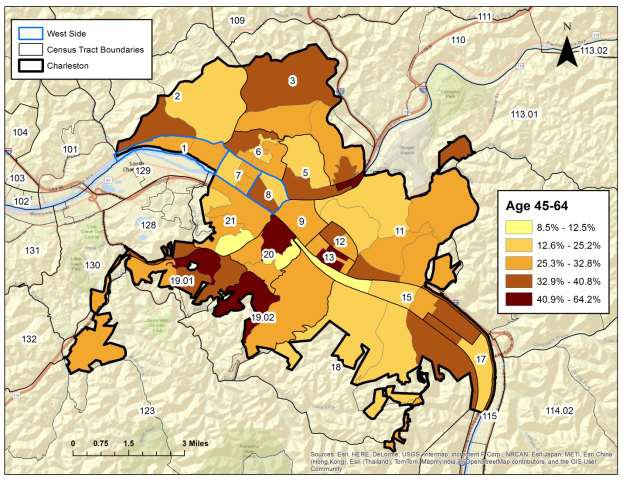


**Figure 9. Population density and distribution of Charleston residents between the ages of 45 and 64, by Block Group**


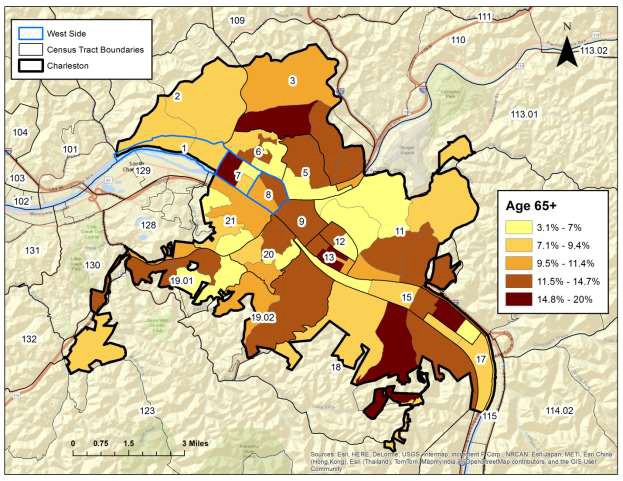


**Figure 10. Population density and distribution of Charleston residents above the age of 65, by Block Group**


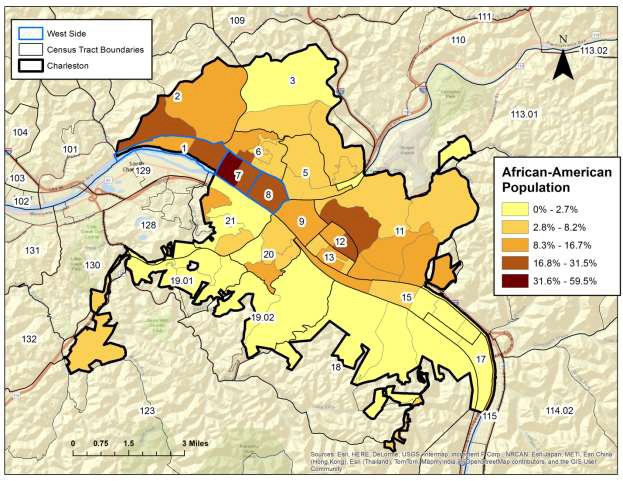


**Figure 11. Distribution of Charleston residents self-identified as “African-American”, by Block Group**


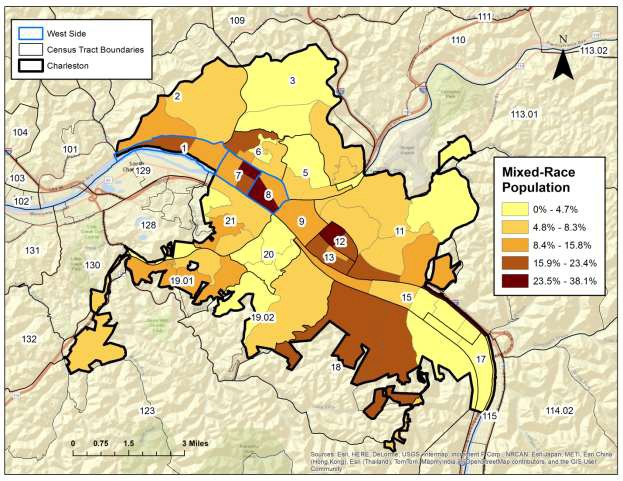


**Figure 12. Distribution of Charleston residents self-identified as “Mixed-Race”, by Block Group**

# Education Trends

To characterize various trends in education achievement, data was collected on middle and high school proficiency scores, high school diploma obtainment rates and geographic distribution patterns, and undergraduate college diploma obtainment rates. Figure 13 shows the location of middle and high schools in the Kanawha County school district. The results of reading and math proficiency scores for both middle and high school attendance zones in the Kanawha County school district are shown in Figures 14 and 15. West Side students attend Stonewall Jackson Middle School and Capital High School. Regarding the 2012-13 middle school proficiency rates, Stonewall Jackson students ranked low in both reading (43.0%) and math (38.3%) proficiency rates when compared to the combined middle and high school proficiency rates for the entire Kanawha County school district (reading, 51.0%; math, 46.1%), as well as state-level rates (reading, 47.2%; math, 42.0%).

Student proficiency rates at Capital High School are more mixed concerning achievement levels when compared to district-level and state-level trends (Figure 15). Reading rates at Capital (50.9%) are identical to district-wide rates (51%) and actually slightly exceed state-level rates (47.2%). Math rates at Capital (40.2%), however, are lower than both district-level (46.1%) or state-level (42.0%) trends.

Figure 16 displays the proportion of residents over the age of 25 that obtained a high school diploma or GED equivalent. The proportion of West Side residents with high school diplomas (72.6%) is only slightly lower than the city-wide percentage (Charleston, 74.9%), but is significantly lower than state-wide levels (West Virginia, 84.4%). Although the percentage difference between the West Side and Charleston as a whole is slight, calculation of a *two proportion Z-test* comparing the West Side and Charleston indicate a significant difference (*Pearson* χ2= 6.244, *df* = 1, *p*=0.013). This result suggests that West Side residents, on average, obtain their diploma at a slightly lower rate than neighboring Charleston residents.

Geographic trends are quite variable across Charleston when the proportions of diploma obtainment are measured against population density. One interesting aspect of these data is the high proportion of West Side residents that have obtained a high school diploma in Block Group 3 of Census Tract 7 (>90%). It is suggested here that this high proportion reflects the fact that this neighborhood is characterized by a substantial elderly population (65+ years of age, see Figure 10) who likely placed a premium on obtaining at least a high school education.

A second way to visualize this data is presented in Figure 17. Here, diploma obtainment was analyzed in ArcMap 10.1 using the Getis-Ord Gi* statistic to determine if geographical clustering of high (hot) or low (cold) diploma obtainment rates could be identified. In other words, the Getis- Ord Gi* statistic helped identify areas within Charleston that have an unusually high, or low, geographic concentration of people with a high school education. As seen in Figure 17, the West Side shows no significant clustering which can be interpreted to mean that no strong geographic trend in the concentration of people with a high school diploma is present in this part of Charleston.

A central grouping of Census Tracts (12, 13, 20), however, is characterized by “cold spots” which indicates a lower-than-expected concentration of people with high school diplomas. These Census Tracts also contain high African-American and mixed-race population centers (Figures 11 and 12). Finally, portions of Census Tracts 123 and 130 were identified as having higher-than-expected concentration of people with high school diplomas. These “hot spot” results, which border the city edge, are difficult to fully evaluate due to the potential that the Getis-Ord Gi* statistic may have been biased due to a lack of neighbors from which the statistic is calculated (Sullivan and Unwin 2010:221).

Figure 18 illustrates the percentage of Charleston citizens that obtained an undergraduate college degree, either a 2-year associates or 4-year bachelors. The proportion of West Side residents that have obtained an undergraduate degree (12.5%) is substantially lower than both the city-wide percentage (Charleston, 24%) and, less so, the state-wide percentage (West Virginia, 19%). Only Block Group 2 of Census Tract 8 is characterized by a relatively high proportion of residents (29%) with an undergraduate degree. In general, this perceived relationship was confirmed as being significant through a *two proportion Z-test* comparing the West Side and Charleston degree rates (*Pearson* χ2= 302.511, *df* = 1, *p*<0.001).

Unlike the geographically non-clustered, or non-patterned, distribution of residents with high school diplomas, Charleston is characterized by strong spatial patterning of residents with undergraduate degrees (Figure 18). Areas south of the Kanawha River, especially Census Tracts 18, 19.01, 19.02, 20, and 21, have high proportions of college educated residents, whereas the opposite holds true for residents in areas north of the Kanawha River and west of Elk River. As discussed below, high rates of college diploma obtainment, when mapped by Block Group, positively correlates to areas of high median income levels.


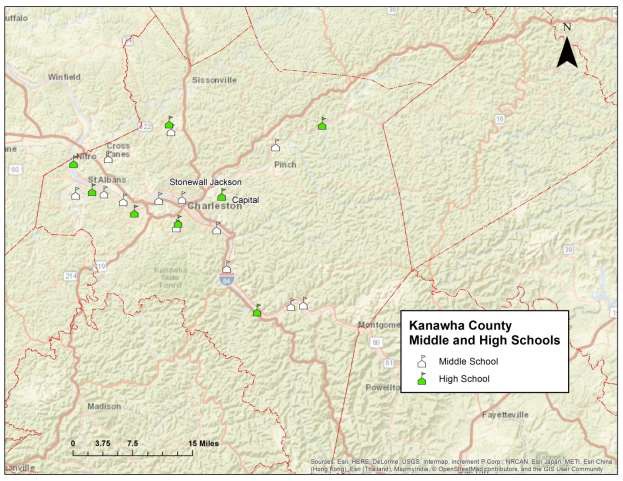


**Figure 13. Location of middle and high schools within the Kanawha County school district. Stonewall Jackson Middle School and Capital High School are labeled and discussed in text.**


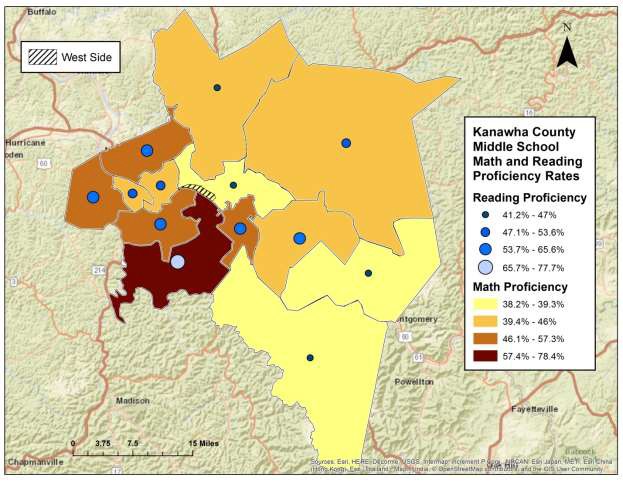


**Figure 14. 2012-13 middle school math and reading proficiency rates per school attendance zone in Kanawha County, West Virginia. Percentages represent proportion of students that scored at, or above, mastery in reading and math. Attendance zone boundaries derived from the Department of Education’s National Center for Education Statistics (NCES), School Attendance Boundary Survey (SABS) 2013-2014. School performance data derived from the West Virginia Department of Education website (https://wvde.state.wv.us/boe/) for the 2012- 13 academic year.**


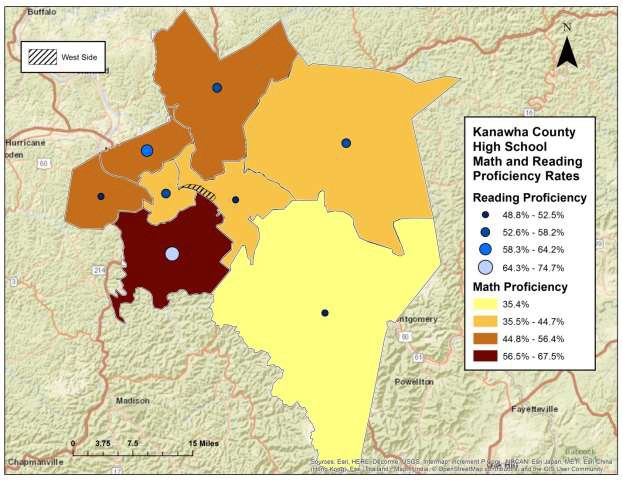


**Figure 15. 2012-13 high school math and reading proficiency rates per school attendance zone in Kanawha County, West Virginia. Percentages represent proportion of students that scored at, or above, mastery in**

**reading and math. Attendance zone boundaries derived from the Department of Education’s National Center for Education Statistics (NCES), School Attendance Boundary Survey (SABS) 2013-2014. School performance data derived from the West Virginia Department of Education website (https://wvde.state.wv.us/boe/) for the 2012- 13 academic year.**


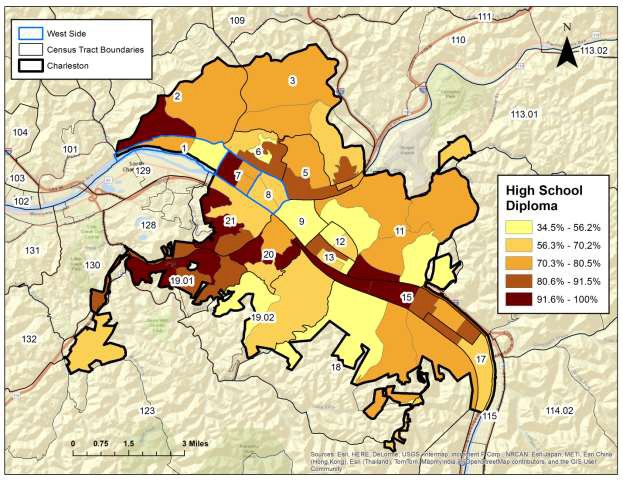


**Figure 16. Distribution of residents above the age of 25 who obtained a high school diploma, or GED equivalent, by Block Group**


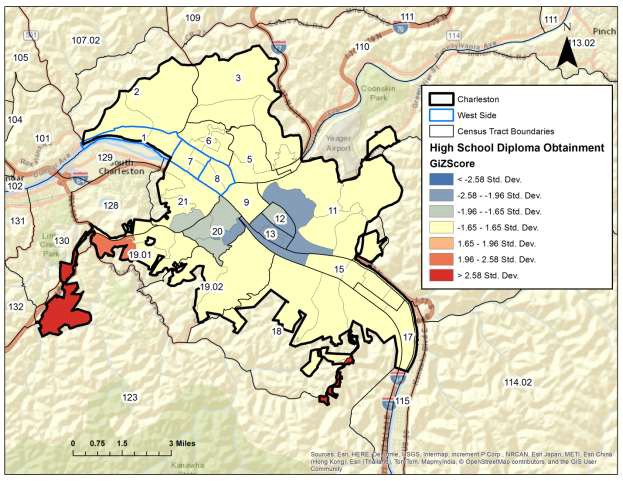


**Figure 17. Results of the Getis-Ord Gi* statistic (standard deviation) calculation showing the distribution of “hot” and “cold” spots across Charleston in relation to high school diploma obtainment, by Block Group**


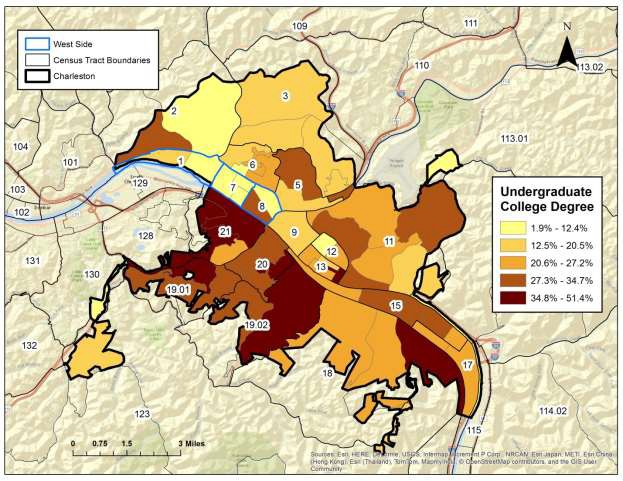


**Figure 18. Distribution of Charleston residents with either a 2-year, or 4-year, college degree, by Block Group**

# Employment

Annual median income values in 2014 inflation-adjusted dollars indicate that West Side citizens have an average median income of $23,197.14 (Figure 19). This income level is $31,993.16 lower than city-wide median income (Charleston, $55,190.30) and $3,824.86 less than state-wide median income (West Virginia, $27,022) (Figure 20). Based on the Census Bureau’s Poverty threshold for 2014, the West Side median income levels are just above, or bordering upon, the poverty line for families containing between two and three individuals, regardless of number of related children within the household (htt[ps://www.c](http://www.census.gov/hhes/www/poverty/data/threshld/%3B)e[nsus.gov/hhes/www/poverty/data/threshld/;](http://www.census.gov/hhes/www/poverty/data/threshld/%3B) Figure 21).

A visual inspection of Figures 19 and 21 suggests that income disparity has strong geographic trends throughout the city as Census Tract 1, 7, 8, 9, 12, and 13 all are characterized by low median incomes and high percentages of people living below the poverty line. The West Side specifically is characterized by especially low median incomes in Census Tract 1, Block Group 1; Census Tract 7, Block Group 1; and Census Tract 8, Block Group 1 (see Figure 19). Moreover, the West Side has over twice as many individuals living below the poverty line (36.6%) than Charleston (18.7%) or West Virginia (18.0%).

To determine if the suggested income disparities were statistically significant for the West Side when compared to the remainder of the city, an *independent t-test* was calculated on group means (Charleston, $55,190.30; West Side, $23,197.14). Test results were significant (*t*=3.245, *df* = 61, *p*=0.002) and demonstrate that, on average, West Side residents earn a substantially lower income than neighboring citizens in other sections of Charleston.

Interesting, when male and female median incomes for the West Side are considered separately, females actually earn a slightly higher median annual income ($16,743) than their male counterparts ($16,398). This trend is opposite of city-wide and state-wide trends where female workers typically earn between 29 and 37 percent less than male citizens (respectively).

Unemployment rates were mapped in Figure 22 for all individuals over the age of 16 that are participating within the civil labor force. According to the U.S. Census Bureau, an “employed” person is defined as:

“Employed includes all civilians 16 years old and over who were either (1) "at work" -- those who did any work at all during the reference week as paid employees, worked in their own business or profession, worked on their own farm, or worked 15 hours or more as unpaid workers on a family farm or in a family business; or (2) were "with a job but not at work" -- those who did not work during the reference week but had jobs or businesses from which they were temporarily absent due to illness, bad weather, industrial dispute, vacation, or other personal reasons. Excluded from the employed are people whose only activity consisted of work around the house or unpaid volunteer work for religious, charitable, and similar organizations; also excluded are people on active duty in the United States

Armed Forces. The reference week is the calendar week preceding the date on which the respondents completed their questionnaires or were interviewed. This week may not be the same for all respondents.” (https://[www.census.gov/glossary/)](http://www.census.gov/glossary/))

Census Tracts 1, 3, 6, 7, 8, 9, 11, 12, and 13 have particularly high rates of unemployment, especially when compared to city-wide (Charleston, 7.3%) and state-wide (West Virginia, 8.2%) trends. The West Side specifically has a combined unemployment rate of 10.3 percent with Census Tract 1, Block Group 1, being characterized by an extremely high rate of 21.1 percent. As with median income, the significantly higher unemployment of the West Side, when compared to the remainder of Charleston, was revealed through a *two proportion Z-test* (*Pearson* χ2= 37.627, *df* = 1, *p*<0.001). Figure 23 shows the breakdown of unemployment status by age group and demonstrates an especially elevated unemployment rate for citizens between the ages of 16 and 24 in Charleston.


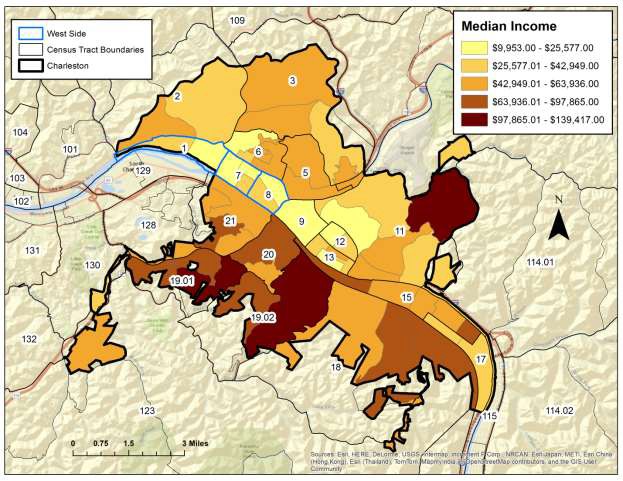


**Figure 19. Medium annual income of Charleston residents, by Block Group**

West Side

Charleston

Average Median Income

West Virginia

0

10000 20000

30000

**Dollars**

40000 50000 60000

**Figure 20. Average median income comparison for West Virginia, Charleston, and West Side**


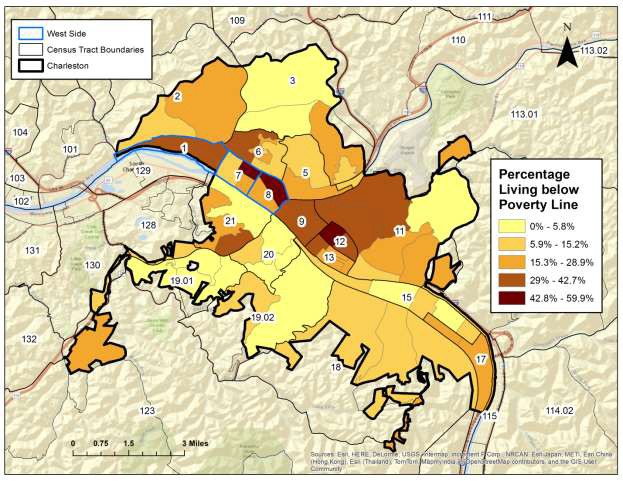


**Figure 21. Percentage of Charleston residents between the ages of 20 and 64 living below the poverty line, by Block Group**


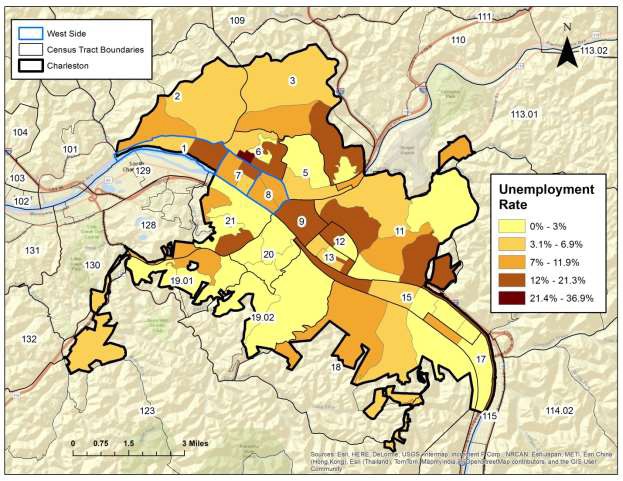


**Figure 22. Unemployment rate for Charleston residents above the age of 16 and participating within the civilian labor force, by Block Group**


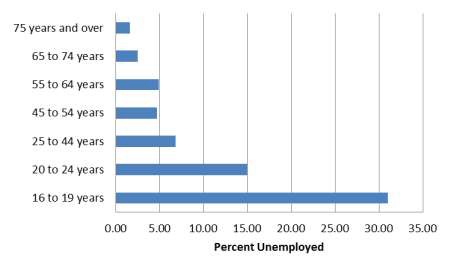


**Figure 23. Civil work force unemployment rate by age group for Charleston**

# Travel Time to Work

Travel time to work can be used as a proxy to measure the accessibility of jobs for people living throughout different communities. Figures 24 through 26 illustrate the distance that Charleston residents travel to work, regardless of mode of transportation (see following section for this variable). Visual inspection of these figures appears to suggest little variation in travel times between West Side residents (<30 minutes, 83%; 30 – 59 minutes, 14%; >60 minutes, 3%) and

Charleston residents (<30 minutes, 87%; 30 – 59 minutes, 10%; >60 minutes, 3%). To better test this visually based assumption, a *two proportion Z-test* was calculated for all three time-period variables (*Pearson* χ2= 52.553, *df* = 2, *p*<0.001). Although a high, thus significant, statistic value was obtained overall, review of standardized residual test scores indicate that only two of the three time-period variables are contributing to this result. These results can be summarized as follows:

- A standardized residual score of +6.7 for the ‘30-59 minute’ variable indicates that a larger- than-expected proportion of West Side workers, as compared to Charleston workers, travel between 30 and 59 minutes to arrive at work.
- A standardized residual score of -2.2 for the ‘<30 minute’ variable indicates that a smaller- than-expected proportion of West Side workers, as compared to Charleston workers, travel less than 30 minutes on their way to work.
- The same proportion of West Side and Charleston workers travel more than one hour to work.

Given that West Side residents significantly rely on public transportation and walking as their primary mode of transportation to work (see below), these data can be interpreted to mean that West Side residents, on average, must select jobs that are closer to their residence than Charleston citizens. This especially appears true in Census Tracts 7, 8, and 9, were walking as the primary mode of transportation to work is markedly high.


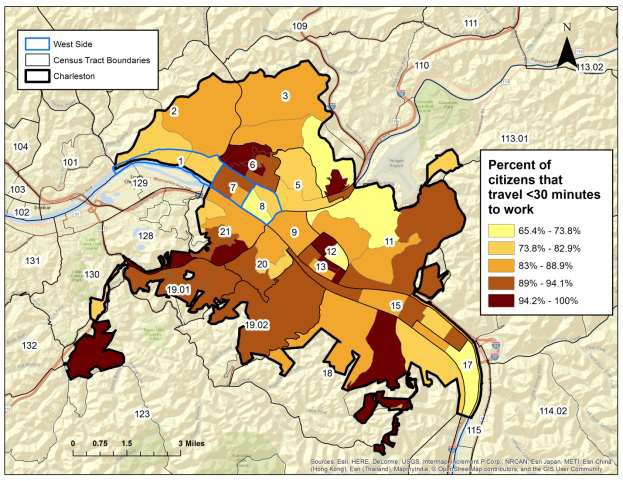


**Figure 24. Map showing percent of citizens that travel less than 30 minutes to work, by Block Group**


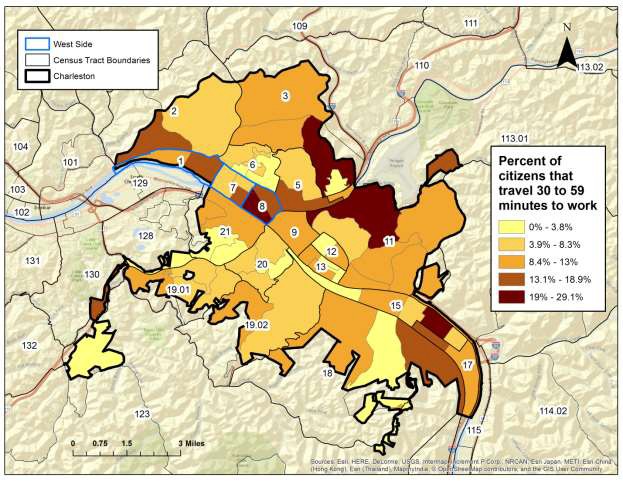


**Figure 25. Map showing percent of citizens that travel between 30 and 59 minutes to work, by Block Group**


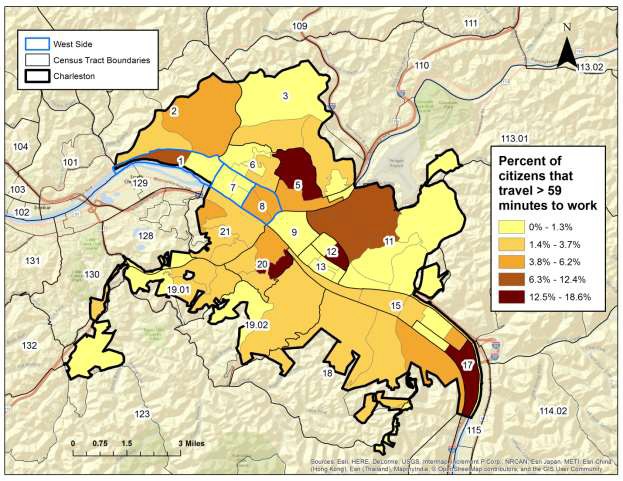


**Figure 26. Map showing percent of citizens that travel greater than 59 minutes to work, by Block Group**

# Primary Mode of Transportation to Work

The manner in which Charleston residents travel to work is discussed in this section. Figures 27 through 29 illustrate the primary mode of transportation for Charleston citizens when traveling for work. This discussion does not include individuals that work from home. Review of the maps suggests a strong geographical patterning with regards to the use of public transportation, walking, and personal vehicle use. A basic dichotomy can be seen in these figures. West Side residents have a high reliance on walking (West Side, 16.7%; Charleston, 5.9%) and public transportation (West Side, 13.4%; Charleston, 4.2%) to get to work, whereas Charleston residents (minus the West Side) are heavily reliant on personal vehicles for transportation. Especially high reliance on walking is seen in Block Group 1 of Census Tract 8 (67%). This high proportion may reflect the fact that this Block Group is situated near the W. Washington Street business district which likely is a key job source for area residents.

To test this initial observation, a *two proportion Z-test* was calculated for all three modes of transportation: walking, public transportation, and private vehicles (*Pearson* χ2= 790.956, *df* = 2, *p*<0.001). As revealed through inspection of standardized residual scores, all three variables are statistically significant which indicates strongly variable transportation trends for residents living in the West Side (public transportation and walking reliant) compared to the rest of Charleston (personal vehicle reliant).


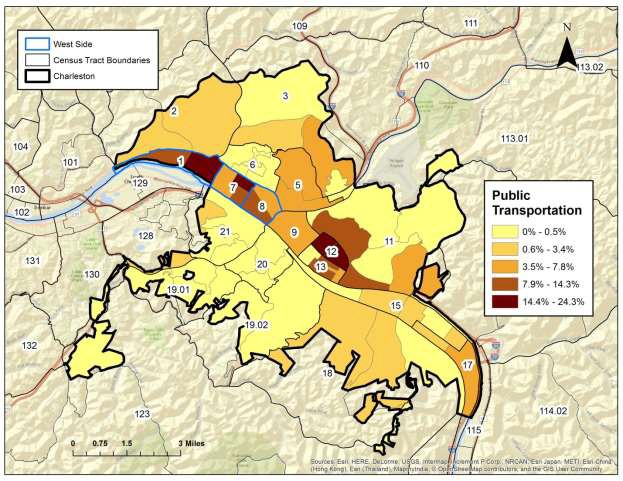


**Figure 27. Percentage of citizens that rely on public transportation (excluding taxi service) as primary mode of transportation for work, by Block Group**


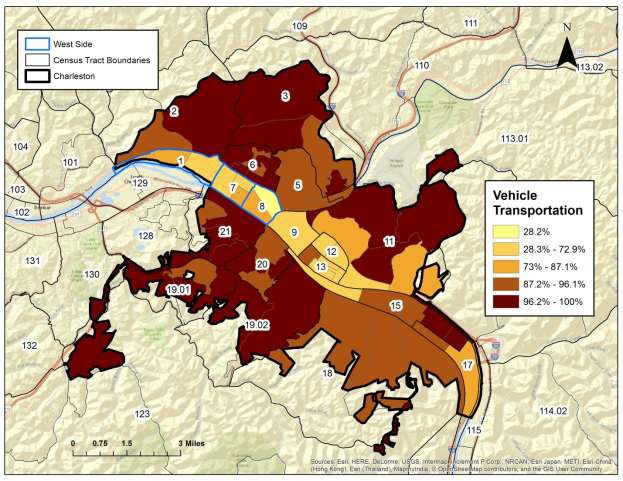


**Figure 28. Percentage of citizens that rely on private motorized vehicles for primary mode of transportation for work, by Block Group**


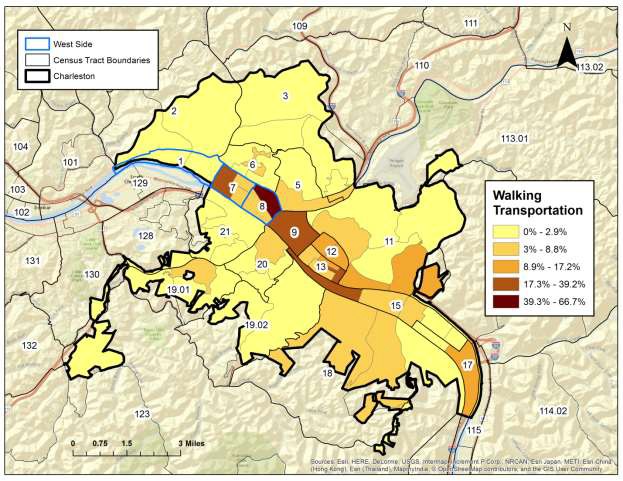


**Figure 29. Percentage of citizens that rely on walking as primary mode of transportation for work, by Block Group**

# Crime Statistics

Crime rates by offense and geographic district are released annually by the Charleston Police Department. This section presents the rate, and distribution, of violent and non-violent crimes throughout Charleston during the 2015 calendar year (Charleston Police Department 2015) (Figure 30). Violent crimes include all instances of rape, murder, and malicious wounding. Non-violent crimes include all instances of robbery, burglary/breaking and entering, larceny, automobile/breaking and entering, and motor vehicle theft. The Charleston Police Department groups crime statistics into one of three districts. The West District includes all city land west of the Elk River and north of the Kanawha River. The East District includes all city land east of the Elk River and north of the Kanawha River. Finally, the South District includes all city land south of the Kanawha River.

As a geographic unit, the West District, which subsumes the West Side, was the location for high rates of both violent and non-violent crimes in 2015. Violent crime in the West District accounts for 52% of all violent crimes committed in Charleston and includes four instances of murder, 27 instances of rape, and 55 instances of malicious wounding. The rate of non-violent crimes also were highest in the West District with over 1000 instances of burglary/breaking and entering, larceny, automobile/breaking and entering, and automobile theft. In the South and East Districts, non-violent crimes represent the remaining 60% of the 2015 crime rates. Finally, the East District had a higher violent crime rate (32%) than the South District (16%), although not nearly as high as the West District (52%).

Calculation of a *two proportion Z-test* (*Pearson* χ2= 16.967, *df* = 2, *p*<0.001) for these three districts confirm the initial observations and show significant patterning in the proportion of violent to non-violent crime among each district. Based on inspection of standardized residual scores, significant differences between districts are accounted for in the over-representation of violent crimes in the West District (+2.4) and the concomitant under-representation of violent crimes in the South District (-3.2). No statistical differences were noted between the districts for non-violent crimes, at least with respect to the proportion of violent to non-violent crimes in this dataset.


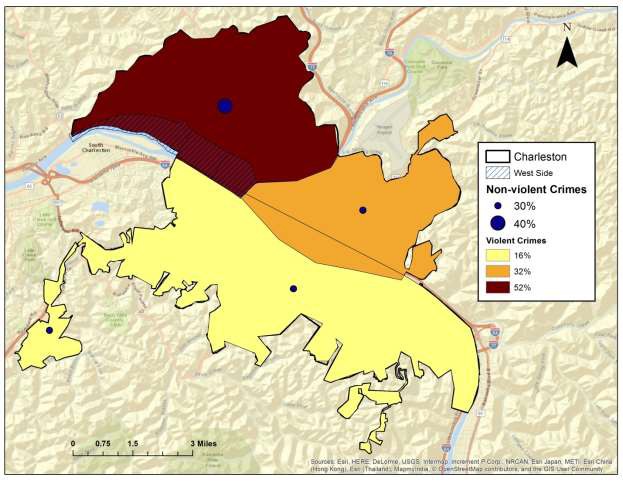


**Figure 30. Distribution of 2015 violent and non-violent crimes for Charleston by crime district**

# Insurance Rates

The proportion of Charleston residents under the age of 65 without health insurance is mapped in Figure 31. These data reflect the U.S. Census Bureau’s 2014 American Community Survey 5-year Estimates and likely does not reflect results associated with the implementation of the 2010 Affordable Care Act. Strikingly, areas both north of the Kanawha River and west of the Elk River display high percentages of residence without insurance, including the West Side which has an uninsured rate of 21 percent. This high rate especially is evident when compared to city-wide (Charleston, 12%) and state-level (West Virginia, 10%) trends. Calculation of a *two proportion Z- test* (*Pearson* χ2= 404.571, *df* = 1, *p*<0.001) further support the suggestion that the geographic distribution of uninsured is patterned and not random.


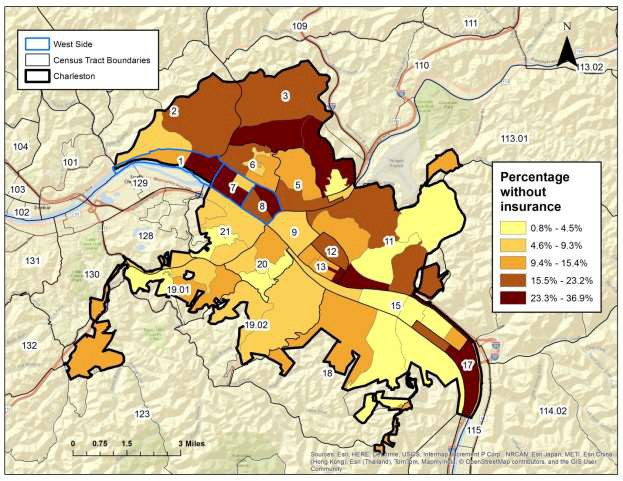


**Figure 31. Percentage of Charleston residents under the age of 65 without healthcare insurance, by Block Group**

# Trends in Housing

Census data on occupancy types and rates illustrates that the West Side is characterized by a comparatively high proportion of vacant buildings; a low proportion of households units occupied by home owners, as opposed to renters; and a low proportion of homes occupied by single families. For this study, “household” refers to all domestic dwellings, either houses or apartment buildings. For the West Side proper (Census Tracts 1, 7, and 8), building vacancy rates are reported as 19 percent which is substantially higher than the city-wide average of 11 percent.

Building vacancy is a notable issue for communities situated within Census Tracts 6, 7 and 8 as rates for these blocks exceed 23 percent (Figure 32). In fact, data suggests that this area possesses the largest geographic concentration of vacant buildings in all of Charleston. To statistically test this apparent association, a *two proportion Z-test* was calculated comparing vacancy rates between the West Side and Charleston. Test results were significant (*Pearson* χ2= 428.253, *df* = 1, *p*<0.001) and indicate that the West Side has a substantially higher rate of building vacancy than other sections of Charleston.

The percentage of household ownership in the West Side (42.0%) appears lower than city-wide (Charleston, 60.1%) or state-wide (West Virginia, 73.0%) trends (Figures 33). This fact is confirmed through calculation of a *two proportion Z-test* (*Pearson* χ2= 480.050, *df* = 1, *p*<0.001) which indicates a comparatively low rate of home ownership for West Side residents. Notably high concentrations of household ownership are found in portions of Census Tracts 20 and 21, just south of the West Side and immediately across the Kanawha River. The opposite trend is observed for renter occupied households which exceeds 60 percent in portions of Census Tracts 7, 8, 9, 12, and 13 (Figure 34).

The distribution of household types – single person, single family, or multiple family - also demonstrates significant spatial patterning across the West Side and Charleston (*Pearson* χ2= 5685.778, *df* = 2, *p*<0.001). Households occupied by a single individual are geographically concentrated in Census Tracts 9, 12, and 13, perhaps representing an abundance of apartment buildings in these tracts (Figure 35). Households occupied by single family units are heavily concentrated along the outer periphery of Charleston (Figure 36). The West Side has a slightly lower percent (46.4%) of households with single family units than Charleston as a whole (54.3%). Multiple family unit housing in the West Side varies little from city-wide trends however (West Side, 6.9%; Charleston, 6.5%) (Figure 37). Census Tract 8 is an exception to this trend as it has an unusually high proportion of multiple family unit housing that suggests that many neighborhood domestic buildings are occupied by two, or more, families.


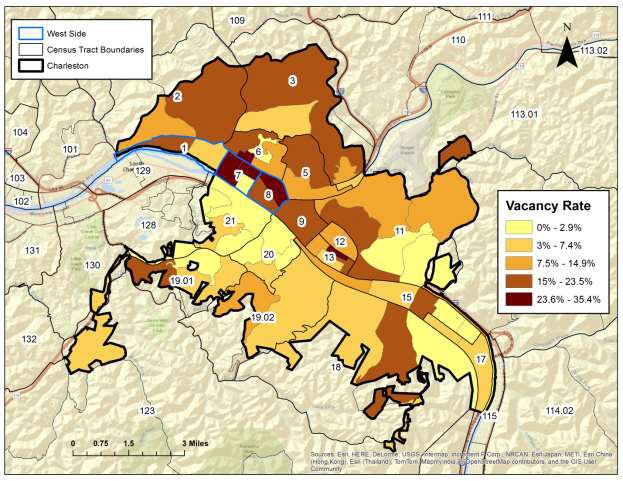


**Figure 32. Percentage of vacant buildings, by Block Group**


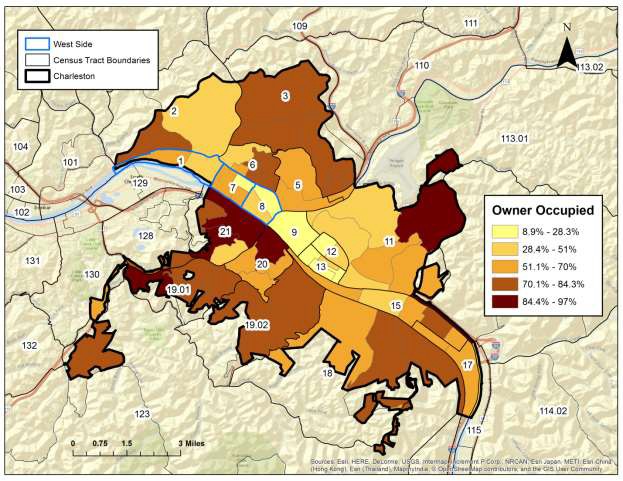


**Figure 33. Percentage of households occupied by owner, by Block Group**


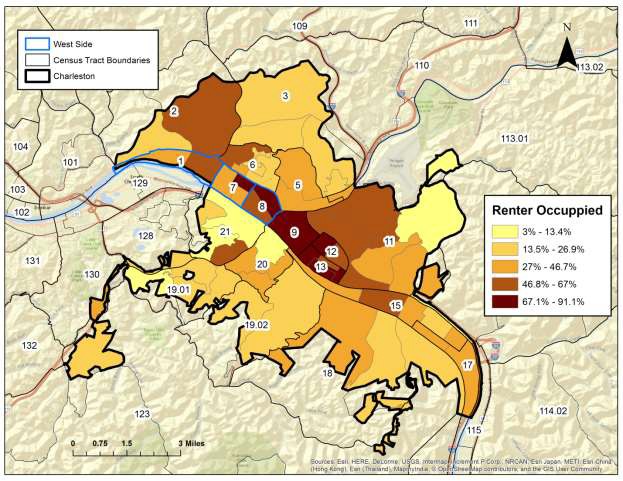


**Figure 34. Percentage of households rented to non-owners, by Block Group**


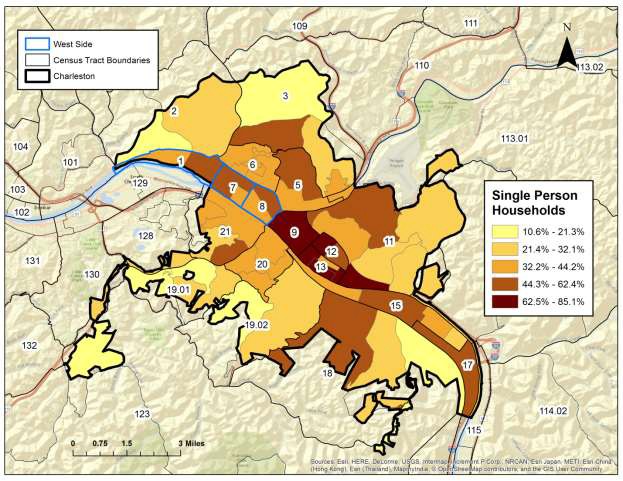


**Figure 35. Percentage of households occupied by a single person, by Block Group**


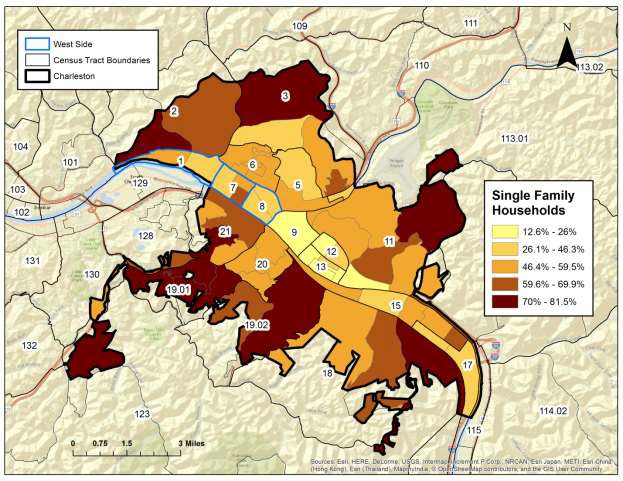


**Figure 36. Percentage of households occupied by a single family unit, by Block Group**


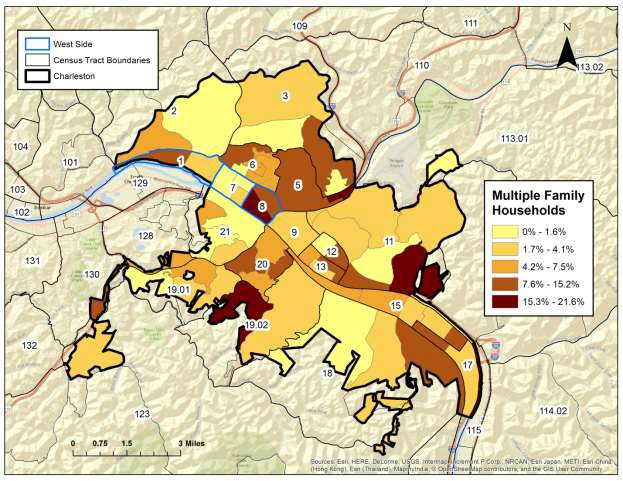


**Figure 37. Percentage of households occupied by two, or more, family units, by Block Group**

# Neighborhood Characteristics

Two aspects of West Side neighborhoods are presented in this section as a way to characterize the living conditions of West Side residents. Specifically, this section discusses the availability of greenspace and public trash cans.

## Greenspace

The accessibility of green space was mapped by Block Group in Figure 38. The availability of urban green space has been linked to numerous general health benefits including reduced stress and depression levels, especially when green space is located within a two mile radius of a person’s residence (e.g., Maas et al. 2006; Ward et al., 2016). For this study, “green space” is defined as any available space containing grass, trees, or other vegetation that allows for both recreational use and aesthetic viewing by the general public. For Charleston, green space includes city- maintained parks, nature preserves, general land, walking trails, school yards and playgrounds, athletic fields, public cemeteries, community centers, and community gardens. Areas subject to dues or membership fees (e.g., YMCA) were not considered as accessible green space. Approximately 1.4 square miles of city-maintained green space is available within Charleston municipal limits.

The distribution of green space across the West Side and Charleston is statistically patterned (*Pearson* χ2= 12385.760, *df* = 1, *p*<0.001). The Block Groups with the highest proportion of green space tend to be situated along the outskirts of the city limits where large parks or walking trails are located including Cato Walking Park in Block Group 1, Census Tract 5; Spring Hill Cemetery in Block Group 3, Census Tract 11; and Blackwell Hiking Trails in Block Group 1, Census Tract

1. With the exception of the Kanawha Blvd Trail or several of the riverside parks, green space within the more urban, high-population, areas of Charleston are restricted to relatively small parcels of land that are not contiguous in spatial distribution. This lack of greenspace is true for the West Side especially.


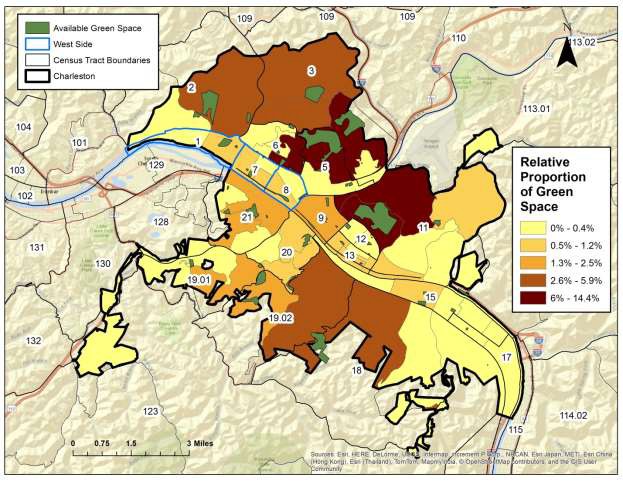


**Figure 38. The location, and relative proportion, of Green Space within Charleston. Data on Green Space locations provided by City of Charleston, Planning Office.**

## West Side City Trash Can Locations

One neighborhood issue repeatedly mentioned when West Side citizens are interviewed is the amount of trash litter scattered about city streets, abandoned parcels, and within neglected yards. Several mention the lack of city provided trash cans as the culprit for this condition since people have few options for disposing of normal, everyday solid waste upon their daily travels. Not only is the amount of litter unsightly and potentially a health hazard, but several studies indicate that it promotes continued littering since individuals view it as a behavior without consequence and socially acceptable (e.g., Cialdini et al., 1990; Reno et al., 1993). Figure 39 maps the location of all city trash cans within the West Side. Trash cans are heavily concentrated along W. Washington Street and, to a lesser degree, along Pennsylvania Street. Importantly, city trash cans are virtually absent from other parts of the West Side where residential housing is abundant.


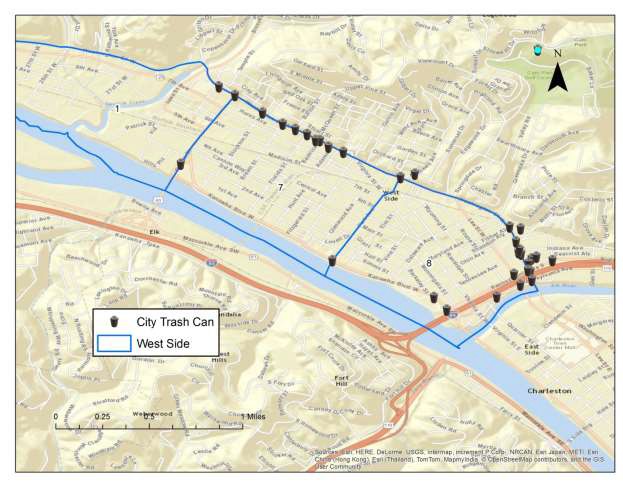


**Figure 39. Distribution of city maintained trash cans within the West Side, specifically Census Tracts 7 and 8. Locational data provided by City of Charleston.**

# Discussion

For this research, 26 indicators of demographic makeup, educational achievement, economic status, crime, health status, green space availability, and housing trends, have been assembled, and analyzed, to characterize potential social inequalities that may exist across the City of Charleston. Specifically, this study has focused on comparing indictors from the West Side -- defined for this study as including city land within Census Tracts 1, 7, and 8 -- to the remainder of the city. This study has collected data from several sources, but most notably from the U.S. Census Bureau’s 2014 American Community Survey 5-year Estimates. As such, analysis and interpretations presented here are a good indicator of current city trends.

As revealed through the geographic mapping and statistical analyses presented earlier, significant inequalities are documented between the West Side and Charleston for all assembled indicators. In general, West Side indicators of demographic, economic, social health, housing, and neighborhood characteristics significantly lag behind those for those of Charleston. The remainder of this report discusses the general trends that have been documented.

The West Side is racially diverse and contains a significantly high African-American and mixed- race population (Figure 40). The high concentration of African-Americans in the West Side has its roots in the historical development of the region extending back to pre-Civil War days. According to Dr. Billy Joe Peyton, Professor of History at West Virginia State University, the land now known as the West Side historically included five large farms owned by prominent local families in the nineteenth century. Prior to the Civil War, these farms included between 70 and 75 African- American slaves. The late nineteenth through middle twentieth centuries witnessed the development of the West Side into an industrial and transportation center for various manufacturing companies. In fact, for much of its early history, the West Side was one of the few places in Charleston where African-Americans could legally purchase property. Accordingly, the West Side developed into an early African-American enclave for the city and was home to many African-American owned businesses.

Indicators also suggest significant inequality between education obtainment rates between the West Side students and the remainder of Charleston. Because middle and high school proficiency rates are collected only by attendance zones, which includes a greater geographic area than the West Side, it is impossible to isolate in these data just how well West Side students are performing in comparison to students from other neighborhoods. It can be stated, however, that math and reading proficiency scores for Stonewall Jackson Middle School, which includes students from the West Side, are noticeably lower than averages for the Kanawha County School District. At the high school level, math and reading scores for Capital High School, which includes West Side students, are not signficantly different than District-wide trends.


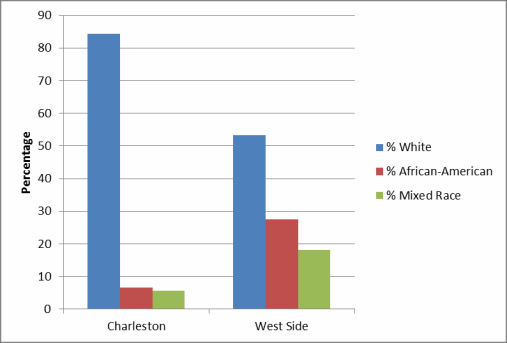


**Figure 40. Comparison of Charleston and West Side racial composition**


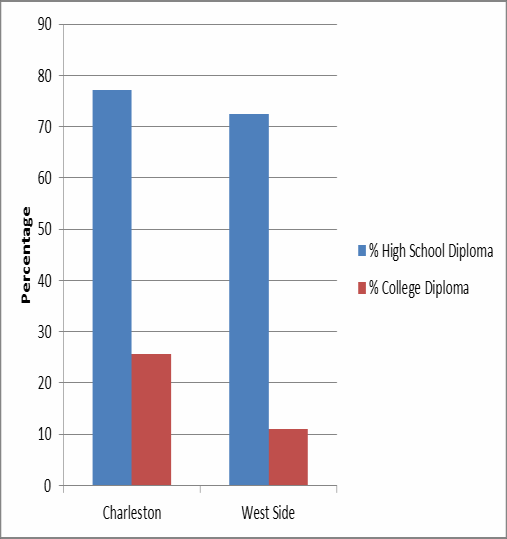


**Figure 41. High school and college diploma obtainment rates for Charleston and the West Side**

The rate at which West Side students obtain high school and college diplomas also is statistically patterned (Figure 41). Although high school and middle school proficiency scores could not be obtained by Block Group, the rate at which people earned a high school or college diploma is available in the U.S. Census Bureau data (see above). The rate at which West Side residents obtained a high school diploma, by age 26, is only slightly lower than the rest of Charleston. Although only slightly lower, this lag is statistically significantly which indicates that, on average, West Side students obtain high school diplomas at a lower rate than other students throughout the city. The disparity between the West Side and Charleston for college degree obtainment is more pronounced and demonstrates a subtantial gap in the number of West Side citizens that earn a college degree (Figure 41).

The fact that fewer West Side residents have high school diplomas and college degrees than other Charleston residents undoubtely attributes to the fact that West Side citizens earn over $30,000.00 less in annual income than Charleston workers (see Figure 20). Aside from education factors, accessibility to job markets also appears to account for some of this income disparity. West Side workers tend to have higher rates of unemployment, are more reliant on walking or public transportation to get to work, and are more likely to have longer commute times to their place of work, than other workers throughout Charleston (Figure 42). The high reliance on walking and public transportation places West Side residents at a competitive disadvantage in their attempts to obtain employment and significantly restricts the geographic radius in which they can effectively search for jobs.

100

90

% Work Travel Time, <30 minutes

80

70

60

% Work Travel Time, 30-59

minutes

% Work Travel Time, >60 minutes

50

40 % Walking Transportation

30

% Public Transportation

20

% Vehicle Transportation

10

0

Charleston

West Side

**Percentage**

**Figure 42. Comparison of Charleston and West Side factors for work travel times and means of work transportation**

Several neighborhood characteristics, social health indicators, and housing trends also demonstrate noticeable disparities between the West Side and Charleston (Figures 43-47). West Side residents

have significantly lower rates of health insurance than other Charleston residents (Figure 43). Moreover, a higher percentage of West Side families live below the Federal Poverty Line when compared to Charleston families. Building vacancy also is a significant problem in the West Side and this area has one of the highest concentrations of abandoned buildings for the entire city (Figure 44). Coupled with the fact that few city-owned trash cans are situated in the West Side (see Figure 39), litter is a constant issue among city streets and at abandoned properties.

When compared to Charleston, the West Side also is characterized by a significantly lower number of homes occupied by owners, a higher number of renters, a higher number of single person households, and a lower number of single family households (Figure 45). The West Side also has significantly less green space than other parts of Charleston (Figure 46). This disparity means that West Side residents have fewer locations where they can exercise, bring children to play, or simply enjoy the outdoors in a safe environment. Finally, crime data for the West Side is difficult to study since it is aggregated into three Districts by the Charleston Police Department and does not match Census Tracts boundaries. The West District includes the West Side and incorporates all areas west of Elk River and north of the Kanawha River. These data show that the West District has significantly higher rates of violent crime than both the East and South Districts combined. Non- violent crimes, in contrast, demonstrate no statistically significant disparity between districts.

40

35

30

25

20

% Civic Unemployment

% below Poverty Line

15

10

5

0

Charleston

West Side

**Percentage**

**Figure 43. Comparison of unemployment rates and percentage of population below poverty line for Charleston and the West Side**


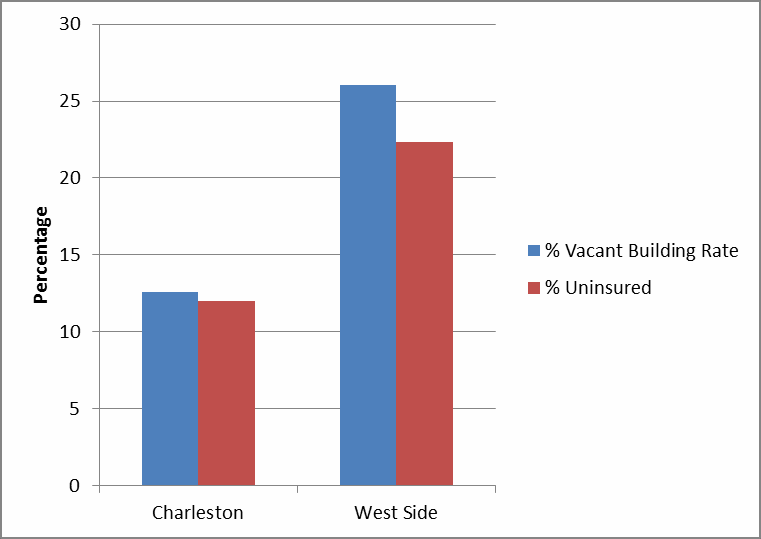


**Figure 44. Comparison of building vacancy rates and percentage of people without health insurance for the West Side and Charleston**


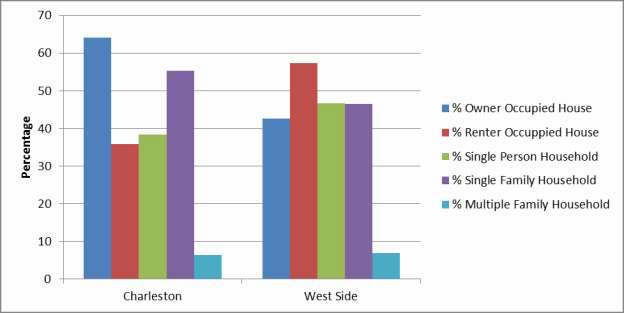


**Figure 45. Comparison of housing trends between West Side and Charleston**


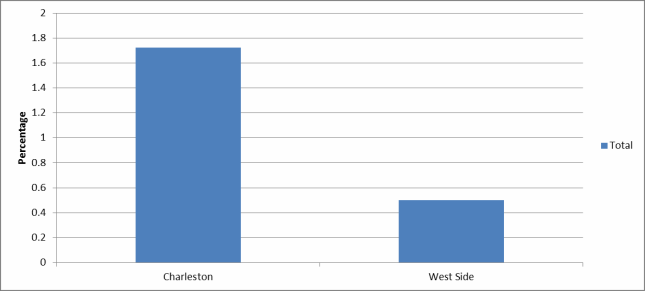


**Figure 46. Comparison of relative percentage of green space by Block Group for the West Side and Charleston**


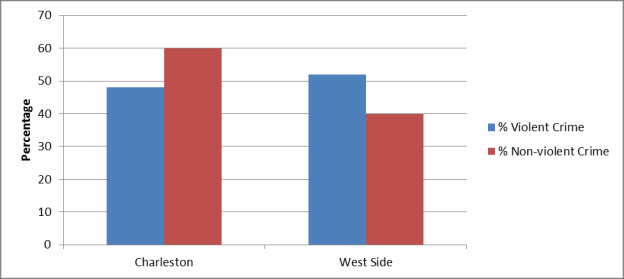


**Figure 47. Comparison of crime statistics between West Side (West District) and the remainder of Charleston (South and East Districts combined)**

## Conclusions

In conclusion, this study has demonstrated that considerable inequality exists between the various indicators measured for residents that live in the West Side and residents that live in the remainder of Charleston. These findings can be summarized as follows:

- - The West Side has a substantial African-American and mixed race population that appears to have historical roots back to the twentieth century.
  - Education indicators suggest that West Side students lag behind their city counterparts in math and reading skill at the middle school level. High school proficiency is more difficult to analyze for West Side students specifically, but based on diploma obtainment rates, there also is a significant lag in the percentage of students that finish high school. College degree obtainment is notably lower for West Side citizens and likely plays a role in why West Side workers earn over 30,000 less in median income than other Charleston workers. This disparity also is at least partially responsible for why West Side residents have high unemployment rates, low insurance rates, and have a significantly higher percentage of the population living below the Federal Poverty Line.
  - West Side workers consistently face obstacles in both finding adequate paying jobs and in traveling to jobs already procured. The high dependence on walking and public transportation restricts the geographic range in which West Side residents can travel for work.
  - West Side neighborhoods are situated in areas characterized by high rates of violent crime and low accessibility to green space for exercise and enjoyment. Building vacancy is a serious issue for the West Side and home ownership is considerably lower than city-wide trends.

# Bibliography

Charleston Police Department. “Charleston Police Department: End of the Year Crime Statistics, 2015.” Unpublished document issued by the Charleston Police Department, Charleston.

Cialdini, Robert B., Raymond R. Reno, and Carl A. Kallgren. "A focus theory of normative conduct: recycling the concept of norms to reduce littering in public places." *Journal of personality and social psychology* 58.6 (1990): 1015.

Maas, Jolanda, et al. "Green space, urbanity, and health: how strong is the relation?" *Journal of epidemiology and community health* 60.7 (2006): 587-592.

O'Sullivan, David, David J. Unwin. *Geographic Information Analysis* (2010). New Jersey: John Wiley & Sons, Inc.

Reno, Raymond R., Robert B. Cialdini, and Carl A. Kallgren. "The transsituational influence of social norms." *Journal of personality and social psychology* 64.1 (1993): 104.

Ward Thompson, Catharine, et al. "Mitigating Stress and Supporting Health in Deprived Urban Communities: The Importance of Green Space and the Social Environment." *International Journal of Environmental Research and Public Health* 13.4 (2016): 44
