# Supplementary material for: Shifting the gaze of the physician from the body to the body in a place: A qualitative analysis of a community-based photovoice approach to teaching place-health concepts to medical students
Source: PLoS One. 2020 Feb 11;15(2):e0228640. doi: 10.1371/journal.pone.0228640 (PMC7012448; doi:10.1371/journal.pone.0228640)
Supplement: S2 File — (DOCX) [file pone.0228640.s002.docx]

**S2 File: Community Sessions**

Session One March 2016 Church-based Community Development Corporation (CDC)

- A five-hour community-based session that included a discussion and presentation on the social determinants of health (SDOH) where the model in Figure 1 was introduced and used to discuss how the features of a place can shape health.
- A historical presentation of issues related to the West Side by the executive director of the Community Development Corporation.
- Break-out and large-group discussions on neighborhoods where medical participants grew up as a point of reference for comparisons to the West Side.
- Narrated community tour led by residents of the West Side.
- Explanation of the photovoice process. Participants were invited to use cell phones or disposable cameras to take photos. Students were cautioned about taking photos as non-members of the community. Taking photos was optional for medical students. All students submitted essays reflecting their observations. All community members agreed to take photos and write captions for their pictures.

Session Two: Early April 2016, County Health Department

- A 2.5-hour session during which medical school participants and community members presented their photos, discussed the significance of the photos, and generated themes based on the photos.
- A presentation on the state code for abandoned buildings by West Virginia University Law School Land-Use Clinic;

Session Three: Late April 2016, County Health Department

- A two-hour session during which medical students and faculty, community members, and community development practitioners participated in video recorded interviews discussing their photos, the significance of the experience, and how it might impact their approach to community involvement and professional role as a physician or community development expert.
