# Supplementary material for: Shifting the gaze of the physician from the body to the body in a place: A qualitative analysis of a community-based photovoice approach to teaching place-health concepts to medical students
Source: PLoS One. 2020 Feb 11;15(2):e0228640. doi: 10.1371/journal.pone.0228640 (PMC7012448; doi:10.1371/journal.pone.0228640)
Supplement: S3 File — (DOCX) [file pone.0228640.s003.docx]

**S3 File: Adaption of Structural Competency Framework**

Researchers originally suggested eight domains and key assessment criteria for a structural competency assessment tool (Bourgois, Holmes, Sue, & Quesada, 2017).

- - - Financial security: resources to live comfortably
    - Residence: a safe, clean/private/quiet/, stable place to sleep and store possessions
    - Risk environments: places to feel safe and healthy
    - Food access: adequate nutrition and access to healthy food
    - Social network: friends, family, or other people who help
    - Legal status: legal problems
    - Education: reading skills, language, and level of education
    - Discrimination: self-reflection on reactions based on a stigma, biases, or negative moral judgments

For this analysis modifications to the eight -domain structural competency assessment tool included the addition of a ninth domain, changes to the definitions of the education and discrimination domains, and the addition of levels of proficiency. First, a ninth domain, structural stigmatization, was added to demonstrate that the identification of a characteristic as negative must occur before discrimination transpires. The term structural stigmatization declares that stigma is more than a one-to-one interaction. In the original structural competency framework, a thing, person, place or characteristic is labeled as negative which then sanctions the use of that stigma in larger systems to condone discrimination or limiting of opportunities. Alternatively, in this nine-domain framework of structural competency structural stigma and discrimination are decoupled and listed separately. Discrimination in the nine domain framework is then defined as the loss of opportunities and resources from systems, institutions and policies based on structural stigmatization (Hatzenbuehler & Link, 2014).

The second modification to the structural competency assessment tool was the expansion of the education domain to include more than observations about the patient’s literacy. The educational domain, in this case, is expanded to include knowledge about the educational system that the patient population has experienced.

**Table 1 Analytical Framework Structural Competency Domains**

| **Domain** | **Definition** |
| --- | --- |
| Financial security | Resources to live comfortably |
| Residence | A safe, clean/private/quiet/ stable place to sleep and store possessions |
| Risk environments | Places where you spend your time each day feel safe and  healthy |
| Food access | Adequate nutrition and access to healthy food |
| Social network | Social network Friends, family, or other people who help you when you need it |
| Legal status | Legal status and or legal problems |
| Education | Reading skills, language, level of education, and knowledge about the educational system |
| Structural Stigmatization | Societal-level conditions, cultural norms, and institutional policies that constrain the opportunities, resources, and  wellbeing of the stigmatized. |
| Discrimination | Able to identify harm or loss of opportunities that could result from a system or institution based on a structural stigma, stereotypical biases, or negative moral judgments. |

SOURCE Lead author’s table defining the nine domains of the structural competency analytical framework.

Last, unlike the original assessment tool, the structural competency framework is given depth by adding four levels of proficiency (Table 2). The original structural competency tool focused on the achievement of three big outcomes: (1) avoidance of a narrow diagnosis of health problems; (2) recognition of systems-level trauma; and (3) contribution to efforts to decrease or at least not make worse social and health inequities (Bourgeois, Orenstein, Ballakur, Mandl, & Ioannidis, 2017). The addition of levels of proficiency recognizes that achieving the three outcomes is a gradual process where avoiding a narrow diagnosis may be an initial step on the way to contributing to efforts to decrease or at least not make health inequities worse

**Table 2 Structural Competency Levels of Proficiency**

| **Level of Proficiency** | **Definition** |
| --- | --- |
| 1st Level of Proficiency | Knowledge about patient that exceeds the individual body to include an understanding of how social and structural systems -the nine domains -of a place shape population health. |
| 2nd Level of Proficiency | Knowledge of external non-medical resources, practices, or policies in the community that address structural issues from the nine domains that contravene the ability of health care practices to improve well-being. |
| 3rd Level of Proficiency | Able to recognize how “I see” that patient and understand how that characterization (individual stigmatization) may be multiplied in systems to result in societal-level, structural stigmatization. |
| 4th Level of Proficiency | Acts as an informed citizen to undo unsuccessful policies, regulations, structures and systems that influence the population health of groups in a place. |

SOURCE: Lead author’s table defining the four levels of proficiency for a practitioner using the structural competency analytical framework.

Bourgeois, F. T., Orenstein, L., Ballakur, S., Mandl, K. D., & Ioannidis, J. P. (2017). Exclusion of elderly people from randomized clinical trials of drugs for ischemic heart disease. *J Am Geriatr Soc, 65*. doi:10.1111/jgs.14833

Bourgois, P., Holmes, S. M., Sue, K., & Quesada, J. (2017). Structural Vulnerability: Operationalizing the Concept to Address Health Disparities in Clinical Care. *Academic Medicine, 92*(3), 299-307. doi:10.1097/acm.0000000000001294

Hatzenbuehler, M. L., & Link, B. G. (2014). Introduction to the special issue on structural stigma and health. *Social Science & Medicine, 103*, 1-6. doi:<https://doi.org/10.1016/j.socscimed.2013.12.017>
