# Supplementary material for: Shifting the gaze of the physician from the body to the body in a place: A qualitative analysis of a community-based photovoice approach to teaching place-health concepts to medical students
Source: PLoS One. 2020 Feb 11;15(2):e0228640. doi: 10.1371/journal.pone.0228640 (PMC7012448; doi:10.1371/journal.pone.0228640)

**S4 File: Photovoice Medical Students**

**1st Level of Proficiency**

The first level of proficiency requires the practitioner to avoid a narrow diagnosis of a health problem by connecting health problems to the SDOH domains in the structural competency framework which is located around the distribution of resources in the place-health model.

Student One

The project’s desire to improve primary prevention of disease is of the utmost importan[ce] in a community with limited resources, both social and financial. If better facilities (food, exercise, recreation, etc.) can be implemented into the heart of the community, than perhaps the residents will utilize them and begin to realize that the ability to take control of their health is a possibility.

Student Two

For me, the most sobering experience of the entire Photovoice project was being able to see the myriad of condemned drug houses in the area. This caused me to think about the people who previously lived there (particularly the children) and what happened to them after they were evicted. In addition, in reference to the condemned houses that are still standing, I thought about the large amount of drug use that most likely occurs behind the doors on a daily basis, in an area that is just minutes to the town I have called home my entire life.

Student Three

Yeah, so on the west side, I’d call it somewhat urban, urban environment. There is not that much green space. There are streets and sidewalks, not perhaps the best place for kids to play. You have to concern yourself with traffic, and more than that, I’ve heard stories of people saying that gunshots ring out quite often. I’m sure when I’m a parent, or even now as a 25 year old, you know, I would not always feel comfortable being out in a place where gunshots are always going off. So I think that forces people indoors and more of a sedentary lifestyle because they can’t go outside and enjoy that aspect of their community.

Student Four

There weren't any areas in the West Side of Charleston that particularly reminded me of where I grew up; in fact it was rather different. The neighborhood I grew up in was very small, with only about 15 houses on a dead-end street. It was a fantastic street to grow up on and was extremely safe and there were always outdoor activities going on with the neighborhood children. We always had parents at home in the neighborhood who would be within eyesight of any children playing outside, or would welcome the youth into their homes for supervision of indoor activities. We were never playing in areas that had items that could be harmful to uninformed children, and we were never running around in areas where dangerous activities would take place. These are all aspects of the community that weren’t very difficult for the adults in the area to help with, but it was helpful that we were located in a small neighborhood. However, these could be potential hurdles for more populated areas to overcome.

**2nd Level of Proficiency**

The second level of proficiency occurs as the practitioner has knowledge of resources that help patients address the SDOH domains that contravene heath care efforts to improve health. There was an absence of photos, captions, and narratives describing what could be done to address problems of West Side community members.

**3rd Level of Proficiency**

The third level of structural competency is evidenced by the practitioner’s ability to see individual level stigmatization within themselves followed by understanding how that becomes structural stigmatization. Many of the insights from students reflected observations about personal biases and stigmatization. It was harder to find evidence of the student’s understanding how personal biases could be multiplied inside systems, structures and institutions resulting in regulations, practices, and policies that restrained opportunities and resources for those who have been stigmatized at a personal level.

Student 1

First, the most important thing we as healthcare providers need to do, is recognize and set aside our own personal biases regarding low-income areas. Too many physicians, as I did growing up, simply ignore these areas and do whatever possible to “tune out” the obvious disparities that exist with the residents who live there. This is absolutely unacceptable; if we want change to occur we have to realize and accept the differences and know that, as members of the healthcare community who devote our lives to assisting those in need, it is 100% our responsibility and duty to care for these people, even if they don’t reach out to us first.

Several students spoke of what they heard about the West Side from others. One third year medical student explained how he reconciled what he heard about the community with observations from touring the community. The student was able to separate out the stigma and find his own lens with which to view the conditions and members of the community.

Student 1

The West Side Outreach program was useful to me in that it allowed me to view a part of Charleston I would’ve probably remained oblivious to otherwise. As a medical student, I’ve heard patients mention this area of the town in a disapproving manner but seeing the living conditions for myself allowed me to form my own thoughts regarding the demographic. As someone who grew up with several friends who lived in similar neighborhoods, it reminded me of them and their relentless desire to improve their living conditions. The sense of community in the area seemed strong, and this is a vital building block to transforming a neighborhood into a better living area. This experience will help shape my professional training in that it provided a reminder of how there are plenty of people in surrounding communities who may not have the means of accessing health care, no matter their living conditions. Not all sick people find their way to the hospital and they need to be kept in mind when it comes to considering the health of the population of a community, city, state, or country.

One student provided an account of how stories they heard about the West Side impacted their views on the community.

Student 1

Throughout my childhood, most of the adults in my community (as well as peers that attended school with me) would constantly advise me to stay away from the West Side due to the high rate of crime and drugs in the area. Therefore, because I was indoctrinated with such a tunnel-visioned view early in life, I never had a desire to go to this area and explore the people and the community…

When asked what they knew about the West Side students admitted to never visiting the West Side despite having grown up near the community.

Student 1

Honestly, not very good things. And what I realized when we went on that tour was even though I grew up in Charleston, I hadn’t actually been on the west side very much, and I think it was due to its reputation and things. I mean I never witnessed any crime or bad activity happening there, but it was kind of like a thing that you don’t really go on the west side unless you need to go on the west side. This is what I grew up being told and knew.

**4^th^ Level of Proficiency**

The 4th level of proficiency is achieved when the practitioner observes and makes informed inferences about the interconnectedness of groups to place through social processes, hierarchies and broader sets of power relationships. Photos, captions, and reflections by students demonstrated an awareness of the role of the educational system and economic development in shaping the health and wellness of West Side residents. Data reflecting what it would take to make systemic and policy level changes varied with some placing responsibility on community members and others asserting the need for partnerships or assistance from decision makers in public and private sectors. The analysis of the data failed to demonstrate that the students understood their civic role in addressing the structural issues either as community members or medical practitioners.

Student One

A concern I have with the project is that the majority of the community has to buy into the concepts of bettering their situation for it to truly be successful. If a significant subset of the population is hesitant to make the sacrifices needed, then this lackadaisical attitude could propagate and become toxic to the goals of those trying to better the area. It will undoubtedly be difficult to get some to dedicate themselves, but if the time is taken to clearly explain the benefits and goals then it will be easier for the community to rally behind the notion.

Student Two

I also think it is imperative for the rest of the city to be involved in this project. The community can have a strong desire to improve itself, but many of the efforts will rely on support from those who reside in different areas of Charleston. The ideas pitched need to initially be heard by the residents of the West Side, but also by those outside of the district who have an interest in helping better the city as a whole.

Several students saw economic development as a key systems level variable in efforts to improve the lives of West Side community members.

Although I was inspired by the multifaceted approach I saw taking place, there was one nagging determent to the areas advancement that I noticed while on the tour. There opportunity to engender enterprise, at an economically profitable rate seemed painfully lacking in the area. A paradigm shift in commerce—that began with the homogenous franchising of American cities; a process that took the business out of the hands of many and into the pockets of few. I noticed this as we drove past several buildings that once seemed to be viable business—be it a grocery store, hardware store, local burger place or convenience store that had all been abandoned—with the only running business left the same ones that dot the exit signs of every American highway. That process affects a location like West Side profoundly because its current residents to not drive into a big city for corporate jobs, or own private offices for their craft---it was a neighborhood built upon local economic trade that had it major source of funds transplanted to a strip mall. This is not to say that West Side is pitted in hopeless fight against an enemy it cannot defeat—it is just to say that the issues present in West Side are not monophasic and not immediately dissolvable. However, with the work of the community, I do feel confident that the West Side is heading in a direction toward progress and change. One that I am proud to have seen and the lessons that I learned during this project will serve me greatly when I become a physician and think about how I want to achieve a transformative impact on a community.

A 4th year medical student documented the need for capitol and human development on the West Side.

I think the first thing I would ask them to undertake, find a way to kind of integrate more businesses into the west side. I might be a little bit biased on that though, just because the friend that I worked with, that’s kind of his big push, is to put more business into the west side to kind of promote it financially from the inside out. I think it would also be interesting to put more work in, put a committee toward helping child health, you know, kind of cutting back and cutting down on abuse risk factors as well as healthy eating. Those just kind of go hand in hand with my interest what I want to do with pediatrics.

Business development as systemic change that could benefit the West Side was depicted in Student Photos 1 and 2.

I believe the story that this photo tells is how the West Side is changing. This massive building has fallen into disrepair (as many people believe that West Side has). But w/ the help of a young entrepreneurs and workers they are rebuilding it and giving it a second chance. This relates to my life because I have close friends who are involved in the

restoration of the building. Some paid money for the actual property while others work inside the building. I think it's a very good metaphor for the "life" of the West Side.

This is a photo of where Kelley's Men Shop used to be (before it relocated to Bridge Row. I wanted to share this photo because I think its interesting metaphor for how businesses are leaving the West Side. To me, the story of this photo shows a business that has been located on the West Side for decades but now lies vacant. I have no idea why they decided to move to Bridge Row, but I find it interesting that a store that sells high end clothing has now relocated to a more upper tier socioeconomic neighborhood after decades of being located on the West Side. This photo resonates w/ my personally because I remember it from my childhood as the super fancy store my dad would take me to. This is the store I went to for prom suit rentals and even was where I had my first suit tailored.

**Photo 1 Stat's Building (corner of Bigley Ave. and Washington St.)**


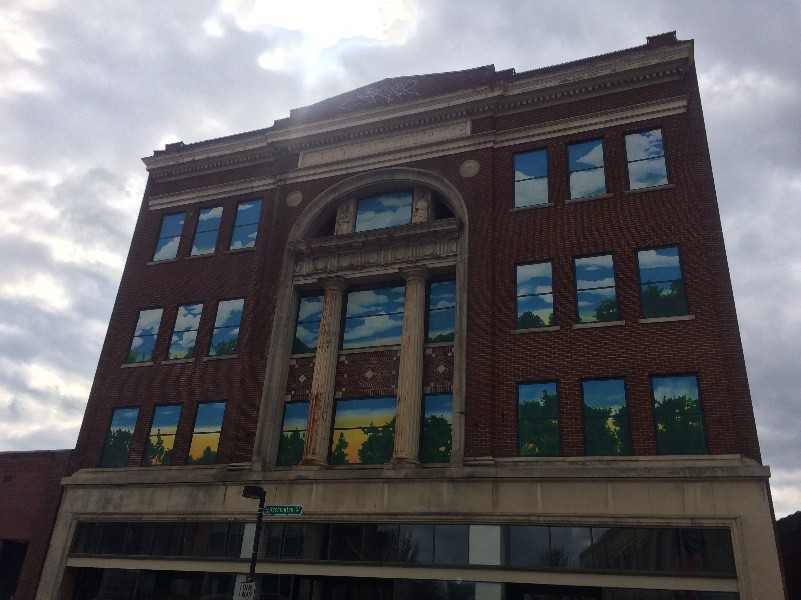


**Photo 2 Former Kelley's Men Shop (Pennsylvania Ave. South)**


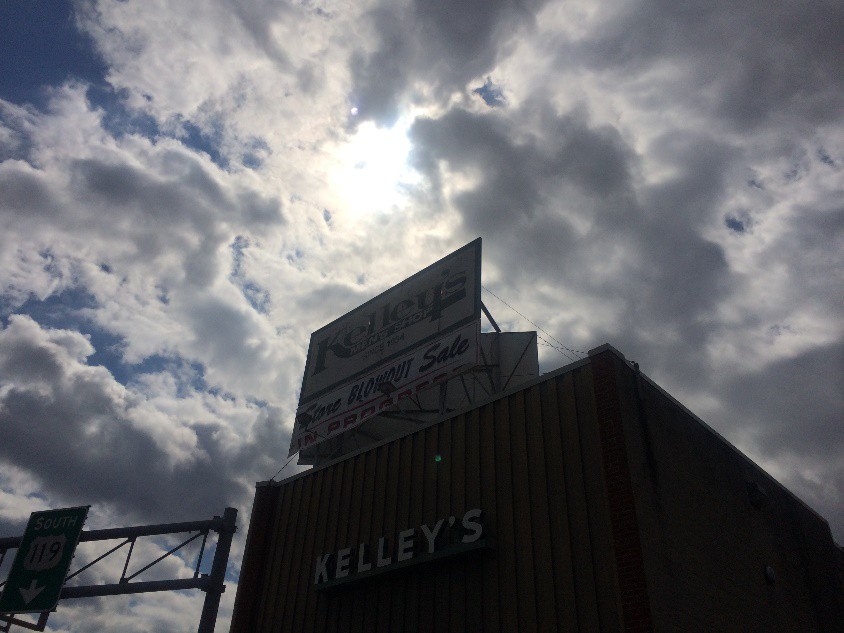


Another 3rd year student demonstrated an awareness of how access to opportunities and resources shaped the lives of West Side residents.

I frequently heard Rev. W’s voice in my head saying that some of the hardest working people he knew were the single mothers in the West Side. I wondered what opportunities (or lack of) they had been given, what choices they may have made that have impacted their current circumstances.

This medical student saw education as a key systems level factor that could improve the lives of West Side community members.

As we drove through the neighborhoods, I noticed the small yards, scarce playgrounds and parks, and no swimming pools. I listened as the poor testing scores of Mary C. Snow West Side Elementary School were discussed, the crime rate of certain areas were noted, and the scarcity of a two-parent household was explained. I also saw families out walking and even caught a glimpse of a few neighborhood kids playing basketball and football in the street on another visit to the West Side. I couldn’t help but wonder about the dinnertime conversations that took place in the houses we passed, about the thoughts that played through the minds of young parents and children at night.

The importance of educational opportunities and resources for the West Side community are depicted in a photo of a neighborhood elementary school. The caption accompanying Photo 3 explains the role that education plays according to the medical student.

Mary C. Snow West Side Elementary School, a relatively new building that serves a population of impoverished students and continues to test poorly in all areas. This photo represents the importance of education in breaking the cycle of poverty, thereby representing hope. It also causes one to wonder how many years it will be before the testing scores begin to change, and to ponder the many factors, beyond education, that need to change to open more doors for children to find a way out of poverty

**Photo 3 Mary C. Snow Elementary School**


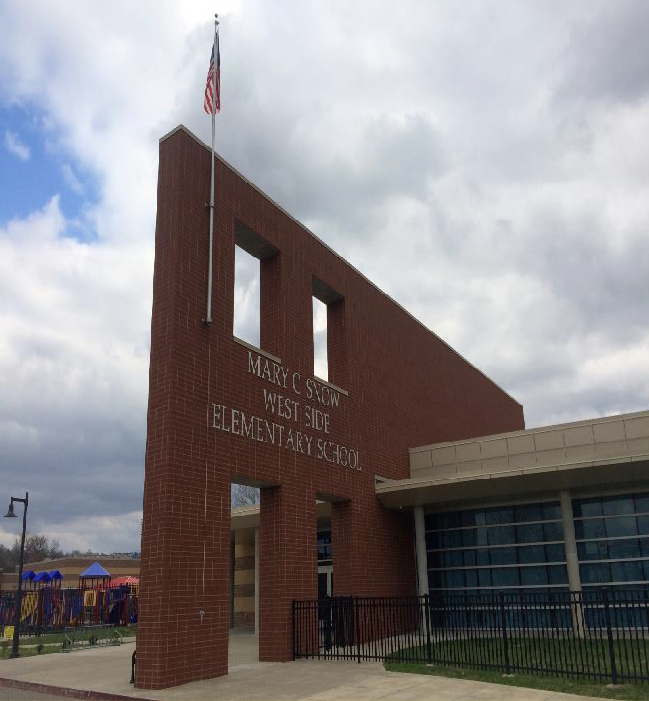

Supplement: S4 File — (DOCX) [file pone.0228640.s004.docx]
