# Supplementary material for: Shifting the gaze of the physician from the body to the body in a place: A qualitative analysis of a community-based photovoice approach to teaching place-health concepts to medical students
Source: PLoS One. 2020 Feb 11;15(2):e0228640. doi: 10.1371/journal.pone.0228640 (PMC7012448; doi:10.1371/journal.pone.0228640)
Supplement: Attachment — Submitted filename: PONE-D-19-22363_reviewer mlr notes.pdf [file pone.0228640.s005.pdf]

PLOS ONE

Shifting the Gaze of the Physician from the Body to the Body in a Place: A Qualitative Analysis of a Community-based Photovoice Approach to Teaching Health-Place Concepts to Medical Students

--Manuscript Draft--

**Manuscript Number:**	PONE-D-19-22363
**Article Type:**	Research Article
**Full Title:**	Shifting the Gaze of the Physician from the Body to the Body in a Place: A Qualitative Analysis of a Community-based Photovoice Approach to Teaching Health-Place Concepts to Medical Students
**Short Title:**	Shifting the Gaze of the Physician: A Qualitative Analysis of a Community-based Photovoice Approach
**Corresponding Author:**	Lauri Andress, PhD, JD, MPH<br>West Virginia University Health Sciences Center<br>Morgantown, WV UNITED STATES
**Keywords:**	Human geography<br>Medicine<br>Linguistics<br>Communications
**Abstract:**	Medical practitioners, trained to isolate health within and upon the body of the individual, are now challenged to negotiate research and population health theories that link health status to geographic location as evidence suggests a connection between health and place. This paper builds an integrated health place model and structural competency framework with nine domains and four levels of proficiency that is utilized to assess a community-based photovoice project's ability to shift the practice of medicine by medical students from the surface of the body to the body within a space. Results demonstrated a set of mixed results on the usefulness of photovoice to attain understanding of place-health based on the use of the integrated place-health model and structural competency framework. The medical student's photovoice data demonstrated that the students achieved structural competency level 1 proficiency and came to understand how health might be connected to place represented by six of the nine domains of the structural competency framework. Understanding at higher levels of proficiency using the community –based photovoice exercise were limited including knowledge of external community resources that address place-health problems, understanding structural stigmatization, and demonstrating the capacity to act as informed citizen to undo unsuccessful structures and systems that influence the population health of groups in a place.
**Order of Authors:**	Lauri Andress, PhD, JD, MPH
	Matthew Purtill
**Additional Information:**	
**Question**	**Response**
**Financial Disclosure**<br><br>Enter a financial disclosure statement that describes the sources of funding for the work included in this submission. Review the submission guidelines for detailed requirements. View published research articles from *PLOS ONE* for specific examples.<br><br>This statement is required for submission	Authors received no specific funding for this work.

Powered by Editorial Manager® and ProduXion Manager® from Aries Systems Corporation

and **will appear in the published article** if the submission is accepted. Please make sure it is accurate.<br><br>**Unfunded studies**<br>Enter: *The author(s) received no specific funding for this work.*<br><br>**Funded studies**<br>Enter a statement with the following details:<br>• Initials of the authors who received each award<br>• Grant numbers awarded to each author<br>• The full name of each funder<br>• URL of each funder website<br>• Did the sponsors or funders play any role in the study design, data collection and analysis, decision to publish, or preparation of the manuscript?<br>• **NO** - Include this sentence at the end of your statement: *The funders had no role in study design, data collection and analysis, decision to publish, or preparation of the manuscript.*<br>• **YES** - Specify the role(s) played.<br><br>* typeset	
**Competing Interests**<br><br>Use the instructions below to enter a competing interest statement for this submission. On behalf of all authors, disclose any competing interests that could be perceived to bias this work—acknowledging all financial support and any other relevant financial or non-financial competing interests.<br><br>This statement **will appear in the published article** if the submission is accepted. Please make sure it is accurate. View published research articles from *PLOS ONE* for specific examples.	Authors have no competing interests.

**NO authors have competing interests**<br><br>Enter: *The authors have declared that no competing interests exist.*<br><br>**Authors with competing interests**<br><br>Enter competing interest details beginning with this statement:<br><br>*I have read the journal's policy and the authors of this manuscript have the following competing interests: [insert competing interests here]*<br><br>* typeset	
**Ethics Statement**<br><br>Enter an ethics statement for this submission. This statement is required if the study involved:<br><br>• Human participants<br>• Human specimens or tissue<br>• Vertebrate animals or cephalopods<br>• Vertebrate embryos or tissues<br>• Field research<br><br>Write "N/A" if the submission does not require an ethics statement.<br><br>General guidance is provided below. Consult the submission guidelines for detailed instructions. **Make sure that all information entered here is included in the Methods section of the manuscript.**	N/A

Powered by Editorial Manager® and ProduXion Manager® from Aries Systems Corporation

**Format for specific study types**<br><br>**Human Subject Research (involving human participants and/or tissue)**<br>• Give the name of the institutional review board or ethics committee that approved the study<br>• Include the approval number and/or a statement indicating approval of this research<br>• Indicate the form of consent obtained (written/oral) or the reason that consent was not obtained (e.g. the data were analyzed anonymously)<br><br>**Animal Research (involving vertebrate animals, embryos or tissues)**<br>• Provide the name of the Institutional Animal Care and Use Committee (IACUC) or other relevant ethics board that reviewed the study protocol, and indicate whether they approved this research or granted a formal waiver of ethical approval<br>• Include an approval number if one was obtained<br>• If the study involved *non-human primates*, add *additional details* about animal welfare and steps taken to ameliorate suffering<br>• If anesthesia, euthanasia, or any kind of animal sacrifice is part of the study, include briefly which substances and/or methods were applied<br><br>**Field Research**<br><br>Include the following details if this study involves the collection of plant, animal, or other materials from a natural setting:<br>• Field permit number<br>• Name of the institution or relevant body that granted permission	
**Data Availability**<br><br>Authors are required to make all data underlying the findings described fully available, without restriction, and from the time of publication. PLOS allows rare exceptions to address legal and ethical concerns. See the PLOS Data Policy and FAQ for detailed information.	Yes - all data are fully available without restriction

A Data Availability Statement describing where the data can be found is required at submission. Your answers to this question constitute the Data Availability Statement and **will be published in the article**, if accepted.<br><br>**Important:** Stating 'data available on request from the author' is not sufficient. If your data are only available upon request, select 'No' for the first question and explain your exceptional situation in the text box.<br><br>Do the authors confirm that all data underlying the findings described in their manuscript are fully available without restriction?	
**Describe where the data may be found in full sentences. If you are copying our sample text, replace any instances of XXX with the appropriate details.**<br><br>• If the data are **held or will be held in a public repository**, include URLs, accession numbers or DOIs. If this information will only be available after acceptance, indicate this by ticking the box below. For example: *All XXX files are available from the XXX database (accession number(s) XXX, XXX.).*<br>• If the data are all contained **within the manuscript and/or Supporting Information files**, enter the following: *All relevant data are within the manuscript and its Supporting Information files.*<br>• If neither of these applies but you are able to provide **details of access elsewhere**, with or without limitations, please do so. For example:<br><br>*Data cannot be shared publicly because of [XXX]. Data are available from the XXX Institutional Data Access / Ethics Committee (contact via XXX) for researchers who meet the criteria for access to confidential data.*<br><br>*The data underlying the results presented in the study are available from (include the name of the third party*	All relevant data are within the manuscript and its Supporting Information files.

and contact information or URL).<br>• This text is appropriate if the data are owned by a third party and authors do not have permission to share the data.<br><br>* typeset	
Additional data availability information:	Tick here if your circumstances are not covered by the questions above and you need the journal's help to make your data available.

manuscript
Click here to download Manuscript Andress place health plos one.docx

Shifting the Gaze of the Physician from the Body to the Body in a Place: A Qualitative Analysis of a Community-based Photovoice Approach to Teaching Health-Place Concepts to Medical Students

Corresponding Author
Lauri Andress, MPH, J.D., Ph.D.
Assistant Professor
Department of Health Policy, Management & Leadership
School of Public Health
West Virginia University Robert C. Byrd Health Sciences Center
laandress@hsc.wvu.edu

Matthew Purtill, Ph. D.
Assistant Professor
Department of Geology and Environmental Sciences
The State University of New York at Fredonia
Matthew.Purtill@fredonia.edu

ABSTRACT

Medical practitioners, trained to isolate health within and upon the body of the individual, are now challenged to negotiate research and population health theories that link health status to geographic location as evidence suggests a connection between health and place. This paper builds an integrated health place model and structural competency framework with nine domains and four levels of proficiency that is utilized to assess a community-based photovoice project's ability to shift the practice of medicine by medical students from the surface of the body to the body within a space. Results demonstrated a set of mixed results on the usefulness of photovoice to attain understanding of place-health based on the use of the integrated place-health model and structural competency framework. The medical student's photovoice data demonstrated that the students achieved structural competency level 1 proficiency and came to understand how health might be connected to place represented by six of the nine domains of the structural competency framework. Understanding at higher levels of proficiency using the community –based photovoice exercise were limited including knowledge of external community resources that address place-health problems, understanding structural stigmatization, and demonstrating the capacity to act as informed citizen to undo unsuccessful structures and systems that influence the population health of groups in a place.

Abstract Word Count 211

Paper Word Count 7932

.

Introduction

Medical practitioners, trained to isolate health within and upon the body of the individual, are now challenged with understanding and integrating research and population health theories that link health status to place. This study provides an example of how medical students may be taught to expand the concept of health beyond the body to include the structures, systems, and institutions where the bodies they treat as patients are located. The paper reports on the development and application of an integrated place-health model and structural competency framework to assess a community-based photovoice project who's goal was to heighten the ability of medical students to understand the health risks associated with places and to shift the practice of medicine from the surface of the body to the body within a place.

Place- Health Concepts

Places where people reside hold the key to the state of health and disease manifest within and on bodies. However, connecting downstream health problems (e.g., chronic diseases) to upstream issues and characteristics of communities is challenging for medical models, pedagogy, and tools (1, 2) (Figure 1). Consequently physicians are rarely prepared to address social and systemic issues because health models and medical training and practices either omit or fail to integrate place-health concepts that could explicate the structural concerns of places where patients reside (1-8).

In her book, *Urban Alchemy*, Mindy Fullilove, a pioneer in psychiatric public health, used systems therapy to demonstrate how population health epidemics, e.g., HIV, drug abuse and addiction, tuberculosis, are best understood as symptoms of disorder within the mechanisms of a larger whole (7, 9). For Fullilove, the systems approach required an understanding of how cities, neighborhoods, and regions are designed, socialized, and managed.

The presence of these downstream health problems suggest that causation and solutions to health problems go beyond individual bodies defined as patients to incorporate a set of systemic institutional, social, and structural issues to bodies now defined as social beings (1, 4). In fact, a foundational premise of health-place research is described as embodiment where the external physical and social worlds are taken in and expressed in human biology (10-16).

Human geography theories add a two-way dimension to embodiment with theories on the social construction of place. Bodies that have been imprinted with external physical and

social worlds are also firstly groups engaged in the social construction of places where they embellish a space with remembrances, imaginings, social relations, and the public assimilation of shared narratives (17-19). Figure 1 demonstrates how the social construction of places by groups and embodiment work together to configure and distribute opportunities in relation to socioeconomic status, race, and ethnicity (16, 20-29).

According to the place-health model in Figure 1, differences in health, social, and economic status at the bottom of the model originate at the top with the cultural toolkit that is attached to a place or region(30). The toolkit is where the construction of place begins as social processes assign meaning to phenomena using a shared set of norms, system of beliefs, narratives, and distinctive spiritual, material, intellectual, and emotional features (31-34). It is through the public assimilation of shared narratives in any given society that groups begin to convert spaces into places (35-37).

In Figure 1, once the cultural toolkit ascribes meaning and a shared narrative, groups use those ideas to assign worth to the social status of others and sanction the distribution of societal opportunities and resources also referred to as the social determinants of health (SDOH). Further, below the cultural toolkit, other influential arenas result from the assigned meanings including (a) the institutions, policies, and systems that govern a place, and (3) the policies, rules and regulations of that place. These mutually reinforcing arenas are the SDOH in a given region that serve to regulate the place in which the citizens live(38, 39).

Finally, the bottom of the model demonstrates the concept of embodiment when it portrays how the SDOH, (i.e., experiences, opportunities, and resources) of a place get under the skin of marginalized groups by three routes: (1) influencing behavior and making behavior harmful; (2) restricting the distribution of and access to key resources; and/or (3) by causing deep seated, chronic anxiety and physiological stress (24, 30, 40-43).

A final principle of the place-health model are the Upstream and Downstream designations to the left of the diagram noting that a society can intentionally use its collective autonomy to work in any or all of these areas: 1) downstream, where illness already exist; 2) upstream on the structural issues including policies, systems, or regulations); or 3) furthest upstream on the cultural toolkit to explore and change the socially constructed, values, beliefs, and narratives that drive the assignment of meaning to group differences and other phenomena (2).

FIGURE 1 HERE

Structural Competency Analytical Framework

A modified structural competency analytical framework is integrated into the place - health model to further actualize mechanisms that explain the association between places and health. For a description of how the structural competency framework was adapted from Bourgeois et al. (2017) see Supplement C. In comparison to cultural competency, which situates individual level symptoms on the bodies of marginalized groups, structural competency extends the diagnosis of individual health to populations and the contributions of institutions, systems, policies, and markets in shaping the health of those groups (1, 44, 45). Structural competency involves the recognition of structural and systemic issues as risk factors that contravene the production of health and individual clinical interventions (1, 4, 44, 46).

Nine domains and key assessment criteria of the structural competency assessment framework are defined in Table 1. Each domain represents a SDOH and pathway through which specific societal resources, systems, and social processes aggravate individual patients' health and shape the health of groups. (46). All the domains are external social and institutional systems constructed by groups that become embodied. The nine domains and their embodiment occur below the assignment of meaning in the place - health model (Figure 1).

Table 1 HERE

The structural competency framework is given depth by adding four levels of proficiency (Table 2). In the modified framework at proficiency level 1 the practitioner avoids a narrow diagnosis of a health problem by connecting the health problem to knowledge about how the place where the patient resides contributes to the health problem seen upon the body. Next, proficiency level 2 has the medical practitioner identify community policy or systems resources that could help the patient if the external problem as one of the nine domains contravene the ability of the patient to be healthy and/or adhere to healthcare interventions. At levels 1 and 2 the practitioner is not expected to understand how places are socially constructed from the cultural toolkit. Rather the practitioner will simply understand that external factors shape health. In the place health model proficiency levels 1 and 2 occur somewhere near the distribution of societal resources described as the structural competency domains (Table 1). The practitioner may see how these

domains shape health but not question or wonder why some groups experience these circumstances more than others.

In the place health model, the vision for proficiency at levels 3 and 4 occur at the top starting at the assignment of meaning, then move into the cultural toolkit followed by work among the policy sectors, institutions, and systems. At level 3 located at the top of the health-place model practitioners begin to consider the origins of what they know about groups in a place and how individual level biases translate into structural stigma. This work occurs within the cultural toolkit with discourse and the construction of narratives that explore the meaning of group differences which have been used to justify the distribution of societal resources. (45, 47). It is at the point of proficiency level 3 that the practitioner should exemplify some understanding of hierarchies and broader sets of power relationships. Acknowledgement of socially constructed places by the practitioner should demonstrate a connection between the visible, material elements of place and the unacknowledged biases, prejudices and inequalities of actors, institutions and systems that shape a space.

The 4th level of proficiency is located around the institutions and polices of the health-place model. At the 4th level practitioners transform into civic members actively contributing to deliberations about the systems, policies, and institutions that play a role in shaping the physical, social, and economic aspects of places that get under the skin to produce population health (18). Political efficacy becomes important as well as the practitioner's ability to merge medical skills with the self as a civic and political being (48, 49).

Methods

Approach

The Institutional Review Board of West Virginia University approved the original study and subsequent analysis of data collected between March and April 2016. A mixed-methods study design was used to implement the original participatory photoanalytic project. The initial project participants (n = 36) were recruited using verbal invitations, distributed fliers, and word-of-mouth among community leaders, families in the community, and medical school faculty. Medical students (n = 10) had to be enrolled in the West Virginia University- School of Medicine, Charleston branch (WVU-SOM-Charleston). Qualitative data (coded interviews,

photos, captions, and reflections) were gathered while demographic, household, economic, neighborhood, educational, and city infrastructure data were plotted and mapped using geographic information systems (GIS) software. The final project included an exhibition and video, “Imagining the West Side: Constructing Health through the Built Environment”. The GIS maps, depictions from the exhibitions, and video may be found in Supplement A. The basis for this paper is the analysis of the qualitative photoanalytic data from the medical students including coded interviews, photos, captions and reflections.

Context

This project took place in Charleston, West Virginia. Specifically, research focused on the West Side neighborhood. GIS maps of all data describing the demographic profile of the community are available (See Supplement A). Nine percent (4,779) of Charleston’s 50,911 residents live in various communities that make up the West Side with demographic data indicating a substantial African-American and mixed-race population (27.4% and 18%, respectively), and a slightly higher ratio of women to men (53% to 47%, respectively). Income disparity has strong geographic expressions. West Side populations have a high percentage of people living below the poverty line (36.6%), especially when compared to Charleston as a whole (18.7%) or West Virginia (18.0%). In 2014 inflation- adjusted dollars, West Side citizens have an average median income of $23,197.14 which is $31,993.16 below Charleston proper ($55,190.30) and $3,824.86 lower than the state median income ($27,022). Charleston Police Department 2015 figures indicate high violent and non- violent crime rates for the West District, which subsumes the West Side.

Recruitment of Study Participants

Project participants (n = 10) were recruited using verbal invitations, distributed fliers, and word-of-mouth among community leaders, families in the community, and medical school faculty. Medical students (n = 10) had to be enrolled in the West Virginia University- School of Medicine, Charleston branch (WVU-SOM-Charleston). Medical faculty (n = 2) and students understood the project as an opportunity to receive an overview of the impact of community factors on health and the perceptions of citizens from the community. Of the ten medical students, seven were men and three were women. Three students were fourth year students, while the remainder where third year students. Medical school participants partnered with West Side community leaders and members and listened to stories and toured the

neighborhood.

Medical students wrote reflections about their participation and how it intersected with their practice of medicine. Students were given the option of taking photos of neighborhood features and writing photo captions. All study participants were informed and agreed that at the close of the project their photos and interviews would be curated into an exhibition and video (Video 1).

Using Community Based Photovoice Research

The use of a community-based photovoie process allowed medical students to acquire knowledge about the place, demonstrate how the knowledge was incorporated into their role as medical practitioner and convey the confluence of these experiences through reflection, narratives, and photos. Community-based participatory research (CBPR) recognizes that external parties to a community are better informed when they work in partnership with community members (50, 51).

Photovoice, considered a form of CBPR, facilitates co-creation and opens up a conversation where community members and external agents may explore person-place dynamics, document contextual strengths, assess complex conditions, and examine policies that influence the lives of the community (52-55).

For this study, the photovoice process included photos with captions from the medical school participants, written reflections, and video recorded interviews. Rather than being directive and leading, the principal researcher used guiding and coaching skills to implement three interactive photovoice sessions for community members and medical students. The three community-based sessions: (1) covered theories on SDOH;(2) engendered an exchange of memories between the community members and medical students/faculty around places where people grew up and lived; (3) explained the photo voice processes; (4) implemented a tour of the community lead by neighborhood members.; (5) included a group exchange to review captured photos; (5) executed a discussion on abandoned buildings and the State Code in West Virginia, and (6) implemented a series of one- to- one video recorded interviews. See Supplement B for a full outline of the community sessions. At the close of the project all participants were involved in at least one of three exhibitions (See Supplement A).

Qualitative Data Analysis

A qualitative approach was adopted to pick up nuanced experiences and more clearly understand the knowledge, attitudes, and beliefs of the medical students (56-58). Between June and September 2018, all materials generated by the medical students were analyzed according to the principles of directed content analysis using the modified structural competency framework (59-63).

In order to replicate interrater reliability, increase the credibility of the study findings, mitigate researcher bias in the data, and diminish the likelihood of misinterpretation, methods of triangulation were used (64). This included review of collected material on at least three occasions by West Side community members along with medical faculty and students (See Supplementary Material). Paper drafts were reviewed by the geographer responsible for maps, and medical school faculty for agreement. Discrepancies were identified, discussed, and resolved.

Results

Illustrative quotes from the medical students will be used to demonstrate how the photovoice project reflected the structural competency framework and health place model. Additional photovoice data including photos, captions, and narratives from the medical students may be found in Supplement D.

1st Level of Proficiency

The first level of proficiency, requires the practitioner to avoid a narrow diagnosis of a health problem by connecting health problems to the SDOH domains in the structural competency framework which is located around the distribution of resources in the place-health model. Knowledge of the place where that patient resides would help the practitioner to connect that problem to the place and not the individual. Medical students engaged in a lecture on the SDOH, a presentation on the socioeconomic history of the community by a local leader, an interactive discussion between students and community members on experiences growing up in their community, followed by a tour of the neighborhood led by members from the community At the 1st level of proficiency the medical students' photovoice data exhibited an understanding of several structural competency domains including: social networks, financial security, residence, risk environments, food access, and education observing a relationship between these factors and the health of the West Side population.

> With all of these adversities, it is not surprising that the West of Side has experienced worse population health outcomes than other communities in the Charleston area…one of the primary barriers to good health on the West Side is the lack of access to various services and resources. Whether it be healthcare facilities, parks, fitness centers, or grocery stores, the distance or the manner in which one must travel in order to reach these locations poses a significant problem to many inhabitants. Poverty is another factor that influences the health of the area. Families may not be able to afford the drugs or services a love one requires.
> Extending beyond the direct healthcare effects, it also limits what choices a person may have…My involvement in this project has led me to think more about the struggles and circumstances of the patients I encounter in the hospital and clinic. I have more appreciation for what they must endure and overcome, and I believe that this opportunity will serve as a reminder going forward that there is more to healthcare than what lies inside a clinic or hospital.

2nd Level of Proficiency

The second level of proficiency occurs as the practitioner has knowledge of resources that help patients address the SDOH domains that contravene heath care efforts to improve health. There was an absence of photos, captions, and narratives describing what could be done to address problems of West Side community members. This lack of photvoice data suggested that the medical students had little knowledge about the nonmedical resources available to help West Side community members. The students may have also failed to grasp the nature of the potential structural problems that restrict the ability of healthcare to improve health outcomes. For example, the West Side is heavily populated by abandoned buildings. The students were intensely curious about the problem. To aide them in considering how to solve a problem of neighborhood blight a discussion on the topic was held with the WVU Law School Land Use Clinic.

3rd Level of Proficiency

The third level of structural competency is evidenced by the practitioner's ability to see individual level stigmatization within themselves followed by understanding how that becomes structural stigmatization. Proficiency. At level 3 the practitioner should exemplify some understanding of hierarchies and broader sets of power relationships.

Many of the insights from students reflected observations about personal biases and stigmatization. It was harder to find evidence of the student's understanding how their personal biases could be multiplied inside systems, structures and institutions resulting in regulations, practices, and policies that restrained opportunities and resources for those who

have been stigmatized at a personal level.

Several students spoke of what they heard about the West Side from others. One third year medical student explained how he reconciled what he heard about the community with observations from touring the community. The student was able to separate out the stigma and find his own lens with which to view the conditions and members of the community.

One student provided an account of how stories they heard about the West Side impacted their views on the community. When asked what they knew about the West Side students admitted to never visiting the West Side despite having grown up near the community.

> First, the most important thing we as healthcare providers need to do, is recognize and set aside our own personal biases regarding low-income areas. Too many physicians, as I did growing up, simply ignore these areas and do whatever possible to "tune out" the obvious disparities that exist with the residents who live there. This is absolutely unacceptable; if we want change to occur we have to realize and accept the differences and know that, as members of the healthcare community who devote our lives to assisting those in need, it is 100% our responsibility and duty to care for these people, even if they don't reach out to us first.

4th Level of Proficiency

The 4th level of proficiency is when practitioners transform into thought leaders and civic members actively contributing to deliberations about the systems, policies, and institutions that play a role in shaping the physical, social, and economic aspects of places. The practitioner should be able to observe and make informed inferences about the interconnectedness of groups to place through social processes, hierarchies and broader sets of power relationships. Photos, captions, and reflections by students demonstrated an awareness of the role of the educational system and economic development in shaping the health and wellness of West Side residents. Data reflecting what it would take to make systemic and policy level changes varied with some placing responsibility on community members and others asserting the need for partnerships or assistance from decision makers in public and private sectors. The analysis of the data failed to demonstrate that the students understood their civic role in addressing the structural issues either as community members or medical practitioners.

Several students saw economic development as a key systems level variable in efforts to improve the lives of West Side community members. A 4th year medical student documented the need for capitol and human development on the West Side. Another 3rd year student demonstrated an awareness of how access to opportunities and resources shaped the lives of West Side residents. This medical student saw education as a key systems level factor that could improve the lives of West Side

community members.

> As we drove through the neighborhoods, I noticed the small yards, scarce playgrounds and parks, and no swimming pools. I listened as the poor testing scores of Mary C. Snow West Side Elementary School were discussed, the crime rate of certain areas were noted, and the scarcity of a two-parent household was explained. I also saw families out walking and even caught a glimpse of a few neighborhood kids playing basketball and football in the street on another visit to the West Side. I couldn't help but wonder about the dinnertime conversations that took place in the houses we passed, about the thoughts that played through the minds of young parents and children at night.

DISCUSSION

Physicians are rarely prepared to address social and systemic issues because health models and medical training and practices either omit or fail to integrate place-health concepts that could explicate the structural concerns of places where patients reside. To determine if medical students could understand and incorporate place-health concepts in their practice of medicine a place health model integrated with a structural competency analytical framework was created and applied to the data generated from a community-based photovoice project. The health place model integrated into the structural competency framework with nine domains, and four levels of proficiency presented a set of metrics useful for evaluating how a community based photovoice project might shift the physician's gaze from the body to the body in a place(Cook et al., 2014b, Keyes and Galea, 2016a). The analysis of qualitative data using the model and framework revealed the following.

First, the medical students were able to use the community-based photovoice process to understand a connection between place and health. Use of the place-health model and structural competency framework to analyze the photovoice data demonstrated a shift in the student's discourse and narratives that described how they conceptualized health beyond the individual body as the reference point. The students were able to work at proficiency level 1 which is located around the distribution of resources or the nine domains in the health – place model (Figure 1).

The student's photovoice data demonstrated very little knowledge of how to address structural, systemic, and policy-based problems as described by Proficiency Level 2. Similarly, at Proficiency level 3 the student's photovoice data reflected individual level stigma learned from family and other relationships but not how the individual level stigmatization multiplied from the individual into the structure of institutions. Understanding hierarchical

structures and power-based relationships was not reflected in the photovoice data which would have demonstrated the basis for achieving proficiency level 3.

Last, the photovoice data of the students did not achieve proficiency level 4 where they merged their physician self into a civic or political role. The photos and narratives did not show that the students had any competency in how to utilize their knowledge, values, and standing on behalf of policies or actions that address and improve the structural issues confronting the West Side community where their patients live.

Using the nine domains of structural competency which are located around the distribution of resources depicted in Figure 1 the analysis of the photovoice data demonstrated that six of nine structural competency domains were identified by the medical students including: financial security, residence, risk environments, food access, social networks, and education. Alternatively, the results of the analysis indicate that the photovoice exercise did not achieve an understanding of the domains of discrimination, legal status, and structural stigmatization by the medical students.

There are several reasons that might explain why the students failed to reach structural competency proficiency beyond level 1 and did not represent specific domains in the photovoice data. The most obvious reason is that the photoanalytic exercise is of limited ability to portray the more complicated ideas of the health –place model and the structural competency framework. Equally, important is that the photovoice exercise did not integrate other opportunities to learn about historical factors on the creation of the community and the socioeconomic changes that occurred over time. The medical students were engaged in a discussion with community members, a tour of the neighborhood lead by community representatives, and a historical, socioeconomic overview by the pastor of the church and community development corporation that cosponsored the project. However, these photovoice elements alone might not have been sufficient to give the students a full understanding of structural, institutional, and systems issues that make up a place.

Second, historical and present day discrimination, power imbalances, and structural stigmatization may not have shown up in the qualitative data from the medical students because these are complex, emotion-charged topics that make them harder to identify, discuss, or admit. In the case of this structural competency framework medical students would be called upon to make the connection between the present-day existence of stigma and the use of the stereotypes to disadvantage groups in a range of socioeconomic opportunities including

housing, healthcare, employment, educational opportunities etc.(Garner, 2017, Morrison and Coates, 2017, Ullucci, 2006). That discrimination is generally rooted in history, still lingers in the present, and requires societies to be responsible for hard-to-define corrective acts makes it all the more difficult to discuss as the notion of a color blind society takes hold making it difficult to make the case for current day discrimination. (Garner, 2017, Morrison and Coates, 2017, Ullucci, 2006). Last, an enhanced photovoice effort, structural competency framework and place health model would still have to recognize and find a way to engage with the entire system of health care pressing down on and controlling how medical practitioners function.

Conclusion

Undoubtedly research confirms that health is the result of the relationship between bodies and places and the structural and social factors that emanate from that place. This study constructed an integrated health place- model and structural competency framework to analyze qualitative data from a community based photovoice exercise that immersed medical students in a place where many of their patients lived. The photovoice project highlighted the importance of narratives and photos as an aide in the development and expression of ideas.

The health-place model integrated into the structural competency framework presented a set of metrics by which to evaluate efforts to shift the physician's gaze to the body in a place. On the other hand, when analyzed using the health place model and structural competency framework, the results demonstrated a set of mixed results on the usefulness of photovoice to shift the practice of medicine by medical students from the surface of the body to the body within a place. The medical student's photovoice data showed an achievement of structural competency at proficiency at level 1 with a failure to achieve proficiency at levels higher than that. The students came to understand how health might be connected to place represented by six of the nine domains of the structural competency framework. Understanding at higher levels of proficiency using the community –based photovoice exercise were limited including: (1) knowledge of external community resources that address the nine domains that make it hard for practitioners to improve health; (2) ability to move from individual stigmatization to structural stigmatization; and (3) capacity to act as informed citizens to undo unsuccessful, structures and systems that influence the population health of groups in a place. Last, an enhanced photovoice effort, structural competency framework and place health model would still have to integrate thinking around the healthcare system that controls how medical

practitioners function.

References

1. Hansen H, Metzl J. Structural Competency in the U.S. Healthcare Crisis: Putting Social and Policy Interventions Into Clinical Practice. Journal of Bioethical Inquiry. 2016;13(2):179-83.
2. Andress L. Using a social ecological model to explore upstream and downstream solutions to rural food access for the elderly. Cogent Medicine. 2017;4(1):1393849.
3. Andress L, Scalise D, Wright JG, Moore SE. Fitting a square peg into a round hole: Perceptions of Appalachian physicians on the incorporation of chronic disease prevention into their practice. Preventive Medicine Reports. 2018;11:216-20.
4. Hansen H, Braslow J, Rohrbaugh RM. From Cultural to Structural Competency-Training Psychiatry Residents to Act on Social Determinants of Health and Institutional Racism. JAMA psychiatry. 2018;75(2):117-8.
5. Fullilove M. Root shock: How tearing up city neighborhoods hurts America, and what we can do about it: Random House Digital, Inc.; 2009.
6. Fullilove MT. Psychiatric implications of displacement: Contributions from the psychology of place. The American Journal of Psychiatry. 1996.
7. Fullilove MT. Urban alchemy: Restoring joy in America's sorted-out cities: NYU Press; 2013.
8. Farmer P. Infections and inequalities: The modern plagues: Univ of California Press; 2001.
9. Fullilove MT. Root shock: How tearing up city neighborhoods hurts America, and what we can do about it: NYU Press; 2016.
10. Bailey ZD, Krieger N, Agénor M, Graves J, Linos N, Bassett MT. Structural racism and health inequities in the USA: evidence and interventions. The Lancet.389(10077):1453-63.
11. Krieger N. Proximal, Distal, and the Politics of Causation: What's Level Got to Do With It? American Journal of Public Health. 2008;98(2):221-30.
12. Krieger N. Public health, embodied history, and social justice: Looking forward. International Journal of Health Services. 2015;45(4):587-600.
13. Krieger N. Theories for social epidemiology in the 21st century: an ecosocial perspective. International journal of epidemiology. 2001;30(4):668-77.
14. Krieger N. Embodiment: a conceptual glossary for epidemiology. Journal of Epidemiology & Community Health. 2005;59(5):350-5.
15. Krieger N. Epidemiology and the web of causation: has anyone seen the spider? Social science & medicine. 1994;39(7):887-903.
16. Petteway R, Mujahid M, Allen A. Understanding Embodiment in Place-Health Research: Approaches, Limitations, and Opportunities. Journal of Urban Health. 2019:1-11.
17. Low SM. Towards an anthropological theory of space and place. Semiotica. 2009;2009(175):21-37.
18. Low SM. Embodied space (s) anthropological theories of body, space, and culture. Space and Culture. 2003;6(1):9-18.

19. Low SM. Spatializing culture: the social production and social construction of public space in Costa Rica. American ethnologist. 1996;23(4):861-79.
20. Case A, Deaton A. Rising morbidity and mortality in midlife among white non-Hispanic Americans in the 21st century. Proceedings of the National Academy of Sciences. 2015;112(49):15078-83.
21. Marmot M. Creating healthier societies. Bulletin of the World Health Organization. 2004;82(5):320-.
22. Marmot MG, Bell R. Action on health disparities in the United States: commission on social determinants of health. Jama. 2009;301(11):1169-71.
23. Singh-Manoux A, Marmot M. Role of socialization in explaining social inequalities in health. Social science & medicine. 2005;60(9):2129-33.
24. Adler NE, Stewart J. Preface to the biology of disadvantage: socioeconomic status and health. Annals of the New York Academy of Sciences. 2010;1186(1):1-4.
25. Auchincloss AH, Gebreab SY, Mair C, Diez Roux AV. A review of spatial methods in epidemiology, 2000–2010. Annual review of public health. 2012;33:107-22.
26. Blanchard AK, Prost A, Houweling TAJ. Effects of community health worker interventions on socioeconomic inequities in maternal and newborn health in low-income and middle-income countries: a mixed-methods systematic review. BMJ Glob Health. 2019;4(3):e001308.
27. Pickett KE, Pearl M. Multilevel analyses of neighbourhood socioeconomic context and health outcomes: a critical review. Journal of Epidemiology & Community Health. 2001;55(2):111-22.
28. Roux AVD. Neighborhoods and health: Oxford University Press; 2018.
29. Swinnerton S. Living in poverty and its effects on health. Contemporary Nurse. 2006;22(1):75-80.
30. Andress L. The emergence of the social determinants of health on the policy agenda in Britain: A case study, 1980--2003: The University of Texas School of Public Health; 2006.
31. Dorfman L, Wallack L, Woodruff K. More than a message: framing public health advocacy to change corporate practices. Health education & behavior. 2005;32(3):320-36.
32. Wallack L, Lawrence R. Talking about public health: Developing America's "second language". American Journal of Public Health. 2005;95(4):567-70.
33. Goffman E. Frame analysis: An essay on the organization of experience1974. null p.
34. Jameson F. On Goffman's Frame Analysis. Theory and Society. 1976;3(1):119-33.
35. Kim J-H. Understanding Narrative Inquiry: The Crafting and Analysis of Stories as Research: SAGE Publications; 2015.
36. Saltmarshe E. Using Story to Change Systems. Stanford Social Innovation Review; 2018.
37. Tsui E, Starecheski A. Uses of oral history and digital storytelling in public health research and practice. Public health. 2018;154:24-30.
38. Friel S, Marmot MG. Action on the social determinants of health and health inequities goes global. Annu Rev Public Health. 2011;32.
39. Marmot M. Social determinants of health inequalities. Lancet. 2005;365.
40. Adler N, Rehkopf D. US disparities in health: descriptions, causes, and mechanisms. Annual review of public health. 2008;29:235.
41. McEwen BS. Stress, adaptation, and disease: Allostasis and allostatic load. Annals of the New York Academy of Sciences. 1998;840(1):33-44.
42. Wallace D, Wallace R. Life and death in Upper Manhattan and the Bronx: toward an evolutionary perspective on catastrophic social change. Environment and Planning A. 2000;32(7):1245-66.
43. Sapolsky RM. The influence of social hierarchy on primate health. Science. 2005;308(5722):648-52.
44. Metzl JM, Hansen H. Structural competency: Theorizing a new medical engagement with stigma and inequality. Social Science & Medicine. 2014;103:126-33.
45. Hatzenbuehler ML, Link BG. Introduction to the special issue on structural stigma and health. Social Science & Medicine. 2014;103:1-6.
46. Bourgois P, Holmes SM, Sue K, Quesada J. Structural Vulnerability: Operationalizing the Concept to Address Health Disparities in Clinical Care. Academic Medicine. 2017;92(3):299-307.

47. Bonilla-Silva E. Rethinking racism: Toward a structural interpretation. American sociological review. 1997:465-80.
48. Clarke HD, Acock AC. National elections and political attitudes: The case of political efficacy. British Journal of Political Science. 1989;19(4):551-62.
49. Gil de Zúñiga H, Diehl T, Ardévol-Abreu A. Internal, external, and government political efficacy: Effects on news use, discussion, and political participation. Journal of Broadcasting & Electronic Media. 2017;61(3):574-96.
50. Blair T, Minkler M. Participatory action research with older adults: Key principles in practice. The Gerontologist. 2009;49(5):651-62.
51. Andress L, Hallie SS. Co-constructing food access issues: Older adults in a rural food environment in West Virginia develop a photonarrative. Cogent Medicine. 2017:1309804.
52. Wang C, Burris MA. Photovoice: Concept, methodology, and use for participatory needs assessment. Health education & behavior. 1997;24(3):369-87.
53. Wang CC, Morrel-Samuels S, Hutchison PM, Bell L, Pestronk RM. Flint photovoice: Community building among youths, adults, and policymakers. American journal of public health. 2004;94(6):911-3.
54. Molloy JK. Photovoice as a Tool for Social Justice Workers. Journal of Progressive Human Services. 2007;18(2):39-55.
55. Novek S, Morris-Oswald T, Menec V. Using photovoice with older adults: some methodological strengths and issues. Ageing and Society. 2012;32(03):451-70.
56. Small ML, editor Lost in translation: How not to make qualitative research more scientific. Report from workshop on interdisciplinary standards for systematic qualitative research Washington, DC: National Science Foundation; 2008.
57. Small ML. How many cases do I need?' On science and the logic of case selection in field-based research. Ethnography. 2009;10(1):5-38.
58. Blanche MT, Blanche MJT, Durrheim K, Painter D. Research in practice: Applied methods for the social sciences: Juta and Company Ltd; 2006.
59. Hsieh H-F, Shannon SE. Three Approaches to Qualitative Content Analysis. Qualitative Health Research. 2005;15(9):1277-88.
60. Krippendorff K. Content analysis: An introduction to its methodology: Sage; 2012.
61. Miles MB, Huberman AM. Qualitative data analysis: An expanded sourcebook: Sage; 1994.
62. Tesch R. Qualitative analysis: Analysis types and software tools: Falmer Press, London; 1990.
63. Humble ÁM. Technique triangulation for validation in directed content analysis. International Journal of Qualitative Methods. 2009;8(3):34-51.
64. Cho JY, Lee E-H. Reducing confusion about grounded theory and qualitative content analysis: Similarities and differences. The qualitative report. 2014;19(32):1.

Figure 1

A Place-Health Model on the Production of Inequities

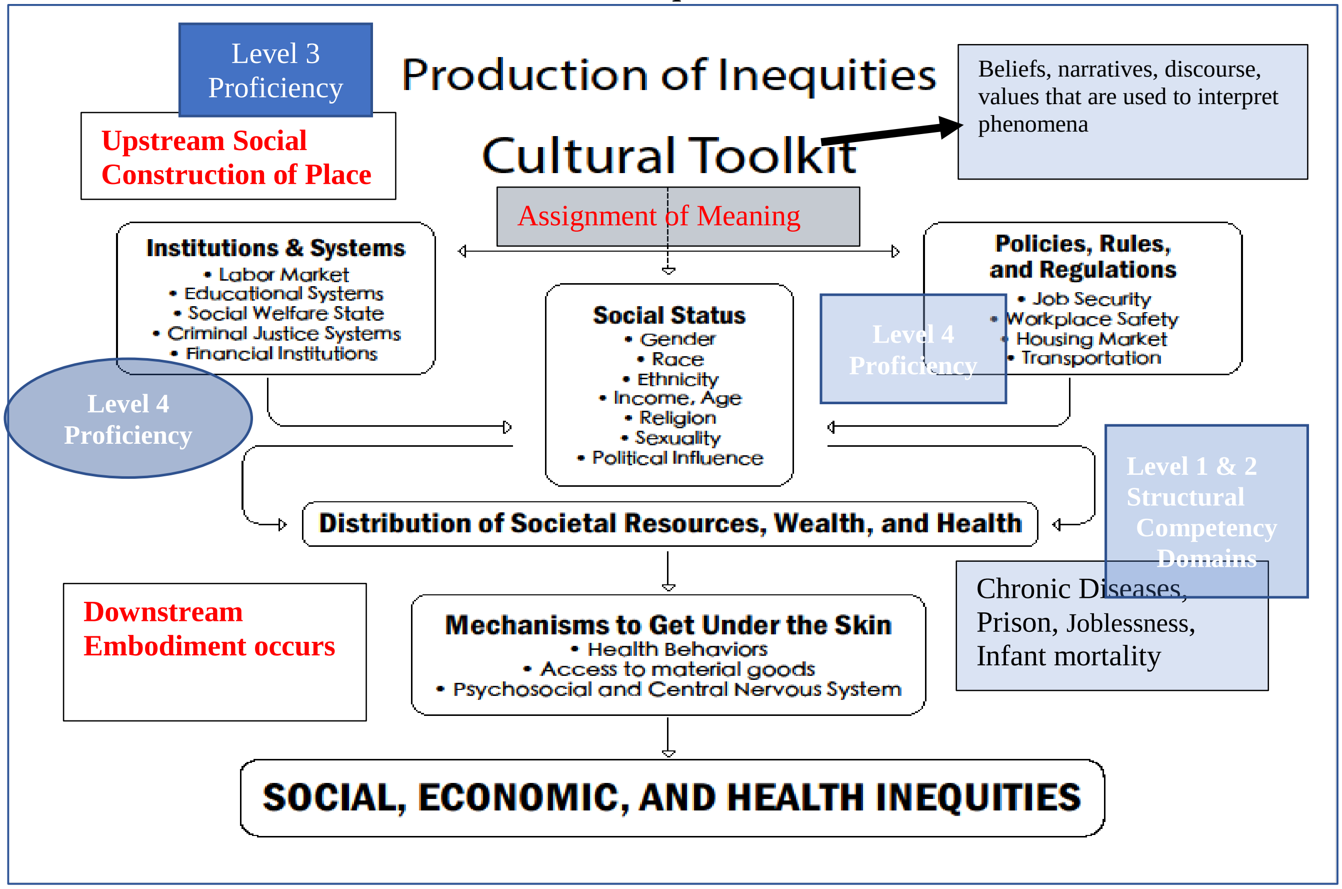

SOURCE Author's analysis of how health and place interact to create social, health and economic inequities.

 Click here to download Figure TAble 1 Domains Structural Comptetency.docx

Table 1 Analytical Framework Structural Competency Domains

**Domain**	**Definition**
Financial security	Resources to live comfortably
Residence	A safe, clean/private/quiet/, stable place to sleep and store possessions
Risk environments	Places where you spend your time each day feel safe and healthy
Food access	Adequate nutrition and access to healthy food
Social network	Social network Friends, family, or other people who help you when you need it
Legal status	Legal status and or legal problems
Education	Education: Reading skills, language, and level of education plus knowledge about the educational system
Structural Stigmatization	Societal-level conditions, cultural norms, and institutional policies that constrain the opportunities, resources, and wellbeing of the stigmatized.
Discrimination	Able to identify harm or loss of opportunities that could result from a system or institution based on a structural stigma, stereotypical biases, or negative moral judgments.

SOURCE Author's table defining the nine domains of the structural competency analytical framework.

Click here to download Figure TAble 2 Levels of Proficiency .docx

Table 2 Structural Competency Levels of Proficiency

Level of Proficiency	Definition
1st Level of Proficiency	Knowledge about patient that exceeds the individual body to include an understanding of how social and structural systems -the nine domains -of a place shape population health.
2nd Level of Proficiency	Knowledge of external non-medical resources, practices, or policies in the community that address structural issues from the nine domains that contravene the ability of health care practices to improve well-being.
3rd Level of Proficiency	Able to recognize how "I see" that patient and understand how that characterization (individual stigmatization) may be multiplied in systems to result in societal-level structural stigmatization.
4th Level of Proficiency	Acts as an informed citizen to undo unsuccessful policies, regulations, structures and systems that influence the population health of groups in a place.

SOURCE Author's table defining the four levels of proficiency for a practitioner using the structural competency analytical framework.

Click here to access/download
Supporting Information
Supplement B Community Sessions.docx

Click here to access/download
Supporting Information
Supplement C strucutural competency framework.docx

supplement D

Click here to access/download
Supporting Information
Supplement D Photvoice Medical Students.docx

Click here to access/download

Supporting Information - Compressed/ZIP File Archive

Supplement A Exhibition.pdf
