# Supplementary material for: Shifting the gaze of the physician from the body to the body in a place: A qualitative analysis of a community-based photovoice approach to teaching place-health concepts to medical students
Source: PLoS One. 2020 Feb 11;15(2):e0228640. doi: 10.1371/journal.pone.0228640 (PMC7012448; doi:10.1371/journal.pone.0228640)
Supplement: Attachment — Submitted filename: Response to Reviewers .docx [file pone.0228640.s006.docx]

Greetings,

The questions raised by the reviewers were very helpful in clarifying concepts and the relationship between ideas and theories used in this paper. The reviewer’s comments were a missing critical analysis that was needed to refine the paper. In the end the reviewers helped us to sharpen our definitions for and distinctions between cultural and structural competency, the description of the place-health model, and the application of the structural competency analytical framework to analyze the photovoice data of the medical students.

Why structural competency and why not cultural competency? We hoped to convey that this is not a one or the other proposition. But rather, that both are needed, may or may not be mutually exclusive, and each covers elements the other does not.

The response to the reviews includes the following items listed below as directed. Further, responses to reviewers provided below are underlined.

A rebuttal letter that responds to each point raised by the academic editor and reviewer(s) uploaded as a separate file and labeled 'Response to Reviewers'.

A marked-up copy of the manuscript that highlights changes made to the original version uploaded as separate file and labeled 'Revised Manuscript with Track Changes'.

An unmarked version of the revised paper without tracked changes uploaded as separate file and labeled 'Manuscript'.

We made an effort to ensure that our manuscript would meet PLOS ONE's style requirements, including those for file naming. We re-checked the manuscript’s details regarding participant consent. Starting in the section, “community based photovoice research” we added more information on the interviewers, their training and characteristics.

We added more details on participant characteristics (age, years of study, etc). We added identifiers to quotations in the manuscript. Finally, we changed the video link in supplement A. The link is connected to a permanent research repository for the library of West Virginia University.

We put tables in our main manuscript leaving supplementary tables as separate "supporting information" files.

**Responses to Reviewer Comments**

**Our responses are underneath each comment and underlined.**

**Reviewer #1: This is brilliant and very important work but it should be re-written so that the** points the authors are trying to make are stated more clearly. The current version tries to convey much too much information, and too many ideas. There is an overabundance of jargon and technical language and almost total lack of discussion of how photography was used or could be used. In addition, it talks about photovoice but does not explain what photovoice is, nor does the body of the article present or discuss specific photographs. The concepts in this paper are very, very important and the authors' approach is groundbreaking and potentially transformative for medical education and practice. I would strongly recommend that this article be accepted with the agreement that the authors work on clarity and paring it down to identify and support the most important points.

We appreciate and agree with many of the observations made within this review. We tried very hard to simplify language and ideas to achieve greater clarity and conciseness. The original project did include photovoice data from community members and medical students. The presentation and discussion of photos is within supplement D. We decided, because of the great amount of content and the desire to focus on the structural competency analytical framework, to use more space in the paper for that purpose.

**Reviewer #2: Physician pv manuscript comments:**

- **The title refers to "Health-Place" but place-health is used throughout, along with other** combinations of the words place and health. Need to be consistent or explain why one is unique to the other.

Agree with reviewer and corrected all instances to be place-health.

- **Citizens instead of citizen on line 52**

Agree with edit and added the word “an” proceeding the word “ citizen “making the sentence s grammatically correct.

- **(In the area of lines 82-90) A consideration for the authors is to think about the language and** training of the medical community. Just as a physician diagnoses the symptomology of the body for diagnosis and direction of a treatment plan (Individual as patient) the same diagnostic process should be seen as viable for assessing the Community as patient when diagnosing the symptomology of the community’s health which can point to potential treatment plans, albeit at a systemic level. A key caveat in this statement is to caution the authors to use this opportunity to educate the health care industry that they should be partnering with public health and NOT feeling the need to be physician of the body AND the physician, if you will, of the physical/social community.

We edited the language slightly in this section so that it does not appear that physicians are required to or capable of address(ing) social/systemic issues. Instead we tried to shape the section to say that physicians should have some knowledge of the social and systemic issues of a place. This is, after all, the first level of four in the structural competency framework.

**On lines 95-97 the authors state” “The presence of these downstream health problems suggests** that causation and solutions to health problems go beyond individual bodies defined as patients to incorporate a set of systemic institutional, social, and structural issues to bodies now defined as social beings”. The beginning of the sentence is fine but then goes off the rails when it concludes with “…to incorporate a set of systemic institutional, social, and structural issues to bodies now defined as social beings”. Now defined as social beings? Please consider rethinking and rephrasing.

We made some adjustments for clarity. The reference to bodies as patients vs. social beings is an important idea meant to convey that the bodies normally encountered in a health setting actually have a history and come from a pace-thus imbuing those bodies with the social, structural and systemic issues attached to the place. Thus, bodies are no longer just the physical corpus but a social entity.

- **How are bodies firstly groups? (lines 101-102).**

Awe added some modifications to this sentence that should help to explain that bodies have a prior existence as part of a group before they are seen by clinicians as individuals in a healthcare setting. Viewed and understood as members of a group helps to ground what is seen within and upon the body within a context and place that is attached to social processes.

- **The authors use the abstract phrasing of ‘cultural toolkit’ without much explanation. Either** rephrase or take the time to explain the use, or the reason for the use, of the phrase. (line 108) What is the role of cultural competence in the cultural toolkit? How does cultural competence contribute to or modify the SDOH and structural competence? (lines 107-108)

These are all relevant questions that are answered as one moves through the paper. Lines 107 to 131 define and provide an explanation of how the cultural toolkit works.

There is no role for cultural competence in the cultural toolkit. The two ideas are easily mixed up because they both unfortunately employ the use of the word “cultural”. The cultural toolkit (a societal concept) is unlike the physician’s traditional understanding of culture which is typically presented as something that an individual possesses that must be understood and used with competence in the clinical experience. In the case of this work, physicians are asked to acknowledge that, because of the cultural toolkit, they are socialized within a family, community, and region followed by a medical pedagogy that shapes the way they think about health. How cultural competence modifies the SDOH and structural competence is only briefly discussed in the section of the paper titled “Structural Competency Analytical Framework where cultural competence is compared with structural competency. In the end one central point is that cultural competence defined as something possessed by other groups cannot acting alone as a concept reduce health inequities.

- **(line 113) ‘convert spaces into places’ is to abstract for a more pragmatic physician audience.** Please consider the rethinking and rephrasing.

We understand that this paper uses theories and concepts from areas of study that may be unfamiliar to physicians. We hope the content proceeding line 113 explains the theory from human geography: *the conversion of spaces into places*. However, that is the overarching point of the paper. In order to more fully address population health, physicians would benefit from understanding the social determinants of health (SDOH). It is the paper’s theory that the use of concepts and theories from other disciplines should help physicians gain this understanding.

- **A general observation regarding the comments above. Some of the language is likely** accessible to the already converted but unsure if it might be a bit too distally worded to resonate with those stuck in a biology based mental frame as many in the health care sector are. A few direct and less flowery statements would help to jar the more resistant readers to new ways of thinking.

We have taken this guidance into consideration and made edits to the paper to make it less flowery. We carefully reviewed the comments from the critique. We re read the relevant section of the paper to determine if our paper needed any changes based on the critique. These changes will show up in track changes. However, as stated, the point of this paper is to demonstrate how the use of nontraditional, health-related concepts and methods from other disciplines can be used to provide physicians with another set of tools by which to work with marginalized groups that experience health inequities.

- **Cultural competency is: "A set of behaviors, policies, and attitudes which form a system or** agency which allows cross-cultural groups to effectively work professionally in situations" and "the integration and transformation of knowledge about individuals and groups of people into specific standards, policies, practices, and attitudes used in appropriate cultural settings to increase the quality of services; thereby producing better outcomes." (lines 140- ) Rethink and rephrase.

We think these descriptions of cultural competency were provided by the reviewer. There are no citations for the definitions provided by the reviewer. Our concept of cultural competency is described in lines 173-175. We did not intend this paper to be a critique of cultural competency. In other drafts of this paper we take on a more full-throated critique of cultural competence as have other researchers. It is a central tenant of this paper that cultural competency is a useful but limited concept. Accordingly, we sought to add on to cultural competency as if the concepts of cultural and structural competency might lie along a continuum to be used as one needs to understand the individual versus the individual within a group situated in a place with contributions from institutions, systems, policies, and markets in shaping the health of those groups. While the reviewer’s description of cultural competency explains how it is used (as policies, a way of behaving) the description of the knowledge and information that informs cultural competency is what is at issue. In the case of cultural competency, the knowledge and awareness are of individuals and groups of people in contrast to structural competency which involves knowledge about and the recognition of structural and systemic issues as risk factors shaping groups.

- **Consider rethinking and rewording lines 137-139 to make more direct and clear;**

This is the language that the reviewer wanted modified: *A modified structural competency analytical framework is integrated into the place -health model of Figure 1 to further actualize mechanisms that explain the association between places and health. For a description of how the structural competency framework was adapted*

Again, these questions are challenging and very helpful in helping us to be clearer. One issue that caused us consternation and perhaps the reviewer is what purpose does it serve to have the place-health model present in this article and then what does it gain us by integrating the place-health model and the structural competency analytical framework?

We returned to the article to try to explain how and why we overlaid and then integrated the structural competency analytical (SCA) framework into the place-health model.

We assert that these models, frameworks and theories help physicians to expand their conception of health from the body to the body in a place impacted by the features of that place.

- **same with lines 150-151.**

The reviewer asked us to make this language more direct and clear: *Each domain represents a SDOH and pathway through which specific societal resources, systems, and social processes aggravate individual patients’ health and shape the health of groups. All the domains are external social and institutional systems constructed by groups that become embodied. The nine domains and their embodiment occur below the assignment of meaning in the place - health model (Figure 1).*

We edited the section to add greater clarity that explains how the SCA framework relates to the SDOH place health model.

- **Can structural competence totally account for this? What is the role of cultural competence** in understanding the connection? (lines 179-181)

Cultural competence makes many claims but has never included nor been able to account for how the features of a place including social, economic, historical and institutional features shape population health. As we have tried to say in our model and framework, we juxtapose structural competence and cultural competence and place them on a continuum that runs from cultural competence to structural competence. In comparison to cultural competence, which is about an individual’s life or being, structural competence is about the features of a place, i.e., systems, institutions, policies, historical facts, that shape a group’s social, historic and economic trajectory in that place.

- **What was the racial and ethnic makeup of participants? Were they native to Charleston or** from elsewhere? What was their socioeconomic background? How did these factors influence their cultural competence in understanding the West Side community?

We described the racial and ethnic makeup of the medical students in the methods section. We did not test for the relationship between the student’s race and ethnicity or birthplace in relation to structural or cultural competency. To comment upon these demographics and any association to structural or cultural competence would be speculation with no grounding in evidence or theories that we reviewed.

- **Photovoice not ‘photovoie’ (line 241)**

This correction was made.

- **Practitioners not ‘practitioner’ (line 242)**

Made a correction to fit the sentence structure by adding the word “a” in the front of the word “practitioner”.

- **(lines 234-238 but also in other areas of manuscript) Photovoice’s ability to highlight the** values and priorities of community members (emic) as opposed to those of the researchers or other outside groups (etic) is a foundational strength of the method, and yet, in this application of photovoice the authors largely chose to retain ‘power’ with the etic/outsider medical community residents to reflect the lived realities of other people. In this way the author’s application is somewhat antithetical to the method’s intended roots. While one can see the author’s aims in their matching of physician residents with community members one is left to wonder why they did not rather choose to let the community members represent their lives themselves through photography, discussion, captioning and public display? At one level this is a fatal flaw of the design. At the same time the authors findings do contribute to the generation of new knowledge for advancing strategies for engendering empathy within the medical profession and the training therein. It would be nice for the authors to at a minimum recognize this limitation of the study being presented and to reflect on an alternative approach that honors and values the power and voice of those so frequently oppressed. Physician residents can still be a part of the process, but a serious balancing of power should be part of the equation.

Please see supplement A where we describe the project more fully and explain how the entire effort included photos taken by both community members and medical students with an aim of integrating the voices of vulnerable groups into public policy decision-making. For this paper we chose to focus upon the photos of the medical students for at least two reasons: (1) the lead researcher has both used and written about photovoice for almost twenty years and has a good understanding of the body of literature on photovoice and gaps in research; and (2) the use of photovoice by medical students could be an effective way to establish greater understanding about how health inequities are created and solutions.

The entire project did serve to highlight the voices of the marginalized community members. Photos, narratives and video interviews from the community members and medical students eventually became an exhibition titled, “ *Imagining the West Side”.* The exhibition explored discrimination that happens when stigmatized groups are marginalized through geography starting with limitations on places where they are allowed to buy property, followed by decades of community neglect and disinvestment, and ending with economic development that transforms the space into a place where the residents are slowly, culturally and economically priced out and displaced by higher income residents. The photos were placed into the exhibition using color coded themes (Photo 1, Supplement A):

- Education/yellow
- Infrastructure/gray
- Hazard/pink
- Economic Prosperity/dark green
- Physical Activity/light green,
- Hope/red
- Housing/blue
- **(line 257) photovoice not ‘photo voice’.**

This correction was made.

- **West Side not ‘West of Side’ (line 298)**

This is a quote from a student’s narrative. It is a common practice to leave quotes as they are written and use brackets to point out any discrepancies or changes to that person’s quote.

- **Loved one not ‘love one’ (line 304)**

This mistake is contained in a medical student’s quote. Any edits of quotes appear in brackets.

- **Why is the work ‘Proficiency’ in the middle of line 328?**

We corrected this mistake.

- **Students’ not student’s (line 331)**

This was corrected.

- **Because this statement is provided on line 341 “When asked what they knew about the West** Side students admitted to never visiting the West Side despite having grown up near the community.” It would seem appropriate to elaborate the thought for the reader. One would assume literature discusses exposure and experience to a growing sense of awareness and potential to budding cultural humility, but it would be good for the authors to elaborate on the point rather than expecting the reader to assume the reason for the statement.

We agree with this observation. This quote and other photovoice data (supplement D) mention never having visited the West Side. We edited the paper in the discussion section including an analysis of the implications of these admissions. In the discussion we highlighted the domains of structural stigma and bias within level 3 proficiency. While the students displayed the ability to recognize their biases none questioned how those biases originated and might result in systemic or institutional biases and stigmatization.

- **On lines 360-362 the authors state: “The analysis of the data failed to demonstrate that the** students understood their civic role in addressing the structural issues either as community members or medical practitioners.” But as a reviewer I am left with the question as to why the authors believe this? Or what their speculative reasoning might be. As a reader one can conclude that they are simply unaware of the duty as seen by the authors, on the other hand the nature of the task seems more aligned with exposure, awareness, and empathy with little discussion of professional role, a larger civic duty or educational challenge to socially structured mores. They do discuss this to a degree in the later part of the paper, but to implicate the methods failure to elicit this action-oriented state may say more about the implementation of the method than anything. Interventions, in this case a unique application of a photovoice-type intervention, can only be expected to measure outcomes that are targeted as process activities of the intervention. To the extent that additional outcomes are a result is interesting but should not be necessarily expected.

We do not disagree with this comment in so far as we understand it. We added a section on limitations of the study to cover failures of the photovoice method to engender elements of the SCA framework in the students. We think this reviewer comment is saying that the photovoice intervention may or may not have been responsible for the failure of the student’s data to demonstrate understanding of their role in addressing structural issues. If this were a quantitative study the results section is where the description of the findings would occur. An explanation as to why the findings turned out this way would occur in the discussion. It is in discussions that we added a section on limitations, In this case (as we say in the discussion) it may be that the data from the students failed to show this outcome because the intervention itself was inadequate, or the intervention process was ineffective, or even because the measurement tool- in this case- the structural competency analytical framework-had not been validated.

- **Capital not ‘capitol’ (line 365).**

This change was made.

- **On line 460 the authors state: “The medical student’s photovoice data showed an** achievement of structural competency at proficiency at level 1 with a failure to achieve proficiency at levels higher than that.” Similar to the earlier comment about the nature of the intervention design, it is challenging to fully expect changes at higher levels without commensurate intervention activities to achieve those aims. Is it possible that incorporating cultural competency might contribute to improving proficiencies at a higher level? (also the ‘than that’ is not needed at the end of the sentence).

First, we made the correction in the sentence structure to eliminate “than that” from the sentence.

Next, we agree that it is difficult to expect achievement of higher levels of proficiency from the medical students. We expressed this conclusion and delineated these same limitations as the reviewer in the discussion section around lines 341 when we discussion limitations.

To summarize, there are at least three reasons why the data might not show evidence that the students achieved higher levels of proficiency: (1) the benefits from photovoice are also its disadvantages where it helps with the articulation of unexpressed ideas, while simultaneously not demanding or having a process that allows for the exploration of more complex ideas and (2) the implementation of the methodology within this study was flawed and thus limited the data; and/or (3) the structural competency analytical framework may not be a valid or reliable tool to measure efforts to train medical students on place-health concepts.

- **As a researcher who values graphics to convey complex thinking and processes this reviewer** appreciates the author’s attempt to convey their ‘Production of Inequities Cultural Toolkit’; however, the organization and flow of the diagram needs a serious rethinking if it is to convey the authors intent. A consideration might be to position the elements within the image into a social ecological frame. As drawn the reader is unclear as to how to process the information provided, where the beginning or end of the model is for the reader, or which elements are grouping labels, and which are constructs. There is too much to unpack to directly rebuild the model, but consideration might be given to enlisting the insights of a fellow researcher who is gifted at graphically displaying complex relationships.

We employed a graphic designer to redraw the figure based on revisions we made. We went into the paper under “Place-Health Concepts” to clarify more fully how to read Figure 1 which is the model. A social ecological model is simply another kind of model that displays the broader factors that shape population health. Most social ecological models are static, showing features that shape health and not how those features interact or flow to shape population health. To support this research and the theories presented which is the work of many years, the features of our model are arranged to follow a path. This is more like a pathway diagram. For example, we take up a fundamental question in the study of the SDOH which is how these factors get under the skin to create good or poor population health. To do this a pathway diagram like ours -unlike a social ecological model- is more effective.
